# Multi-Omics Approach to Elucidate Cerebrospinal Fluid Changes in Dogs with Intervertebral Disc Herniation

**DOI:** 10.3390/ijms222111678

**Published:** 2021-10-28

**Authors:** Anita Horvatić, Andrea Gelemanović, Boris Pirkić, Ozren Smolec, Blanka Beer Ljubić, Ivana Rubić, Peter David Eckersall, Vladimir Mrljak, Mark McLaughlin, Marko Samardžija, Marija Lipar

**Affiliations:** 1 Department of Chemistry and Biochemistry, Faculty of Food Technology and Biotechnology, University of Zagreb, 10000 Zagreb, Croatia; 2 Mediterranean Institute for Life Sciences, 21000 Split, Croatia; andrea.gelemanovic@gmail.com; 3 Clinic for Surgery, Orthopaedics and Ophthalmology, Faculty of Veterinary Medicine, University of Zagreb, 10000 Zagreb, Croatia; bpirkic@vef.hr (B.P.); mlipar@vef.hr (M.L.); 4 Internal Diseases Clinic, Faculty of Veterinary Medicine, University of Zagreb, 10000 Zagreb, Croatia; bla.ljubic@gmail.com (B.B.L.); irubic@vef.hr (I.R.); vmrljak@vef.hr (V.M.); 5 Institute of Biodiversity, Animal Health & Comparative Medicine, College of Medicine, Veterinary Medicine and Life Sciences, University of Glasgow, Glasgow G12 8QQ, UK; David.Eckersall@glasgow.ac.uk (P.D.E.); mark.mclaughlin@glasgow.ac.uk (M.M.); 6 Reproduction and Obstetrics Clinic, Faculty of Veterinary Medicine, University of Zagreb, 10000 Zagreb, Croatia; smarko@vef.hr

**Keywords:** cerebrospinal fluid, dog, integromics, intervertebral disc herniation, metabolomics, proteomics, personalized medicine

## Abstract

Herniation of the intervertebral disc (IVDH) is the most common cause of neurological and intervertebral disc degeneration-related diseases. Since the disc starts to degenerate before it can be observed by currently available diagnostic methods, there is an urgent need for novel diagnostic approaches. To identify molecular networks and pathways which may play important roles in intervertebral disc herniation, as well as to reveal the potential features which could be useful for monitoring disease progression and prognosis, multi-omics profiling, including high-resolution liquid chromatography-mass spectrometry (LC-MS)-based metabolomics and tandem mass tag (TMT)-based proteomics was performed. Cerebrospinal fluid of nine dogs with IVDH and six healthy controls were used for the analyses, and an additional five IVDH samples were used for proteomic data validation. Furthermore, multi-omics data were integrated to decipher a complex interaction between individual omics layers, leading to an improved prediction model. Together with metabolic pathways related to amino acids and lipid metabolism and coagulation cascades, our integromics prediction model identified the key features in IVDH, namely the proteins follistatin Like 1 (FSTL1), secretogranin V (SCG5), nucleobindin 1 (NUCB1), calcitonin re-ceptor-stimulating peptide 2 precursor (CRSP2) and the metabolites N-acetyl-D-glucosamine and adenine, involved in neuropathic pain, myelination, and neurotransmission and inflammatory response, respectively. Their clinical application is to be further investigated. The utilization of a novel integrative interdisciplinary approach may provide new opportunities to apply innovative diagnostic and monitoring methods as well as improve treatment strategies and personalized care for patients with degenerative spinal disorders.

## 1. Introduction

Lower back pain (LBP) is a common disorder with a lifetime prevalence over 70% in the global human population, causing major human health problems and extensive health care utilization [1]. LBP may be caused by degenerative spinal disorders (intervertebral disc damage—herniation or fractures), vertebral infections, or even cancer [2]. Although experimental animals have been used to study degenerative spinal disorders, according to 3R (reduction, refinement and replacement) principles, dogs are reported as the most suitable translational model for biomechanical studies and surgical procedures of the spine and related pathophysiological processes [1,3,4]. In dogs, intervertebral disc herniation (IVDH)-related diseases are the most common cause of spinal cord injury and sensory deficiency [1]. The canine intervertebral disc (IVD) is an elastic structure located between the bony vertebrae and is responsible for the stability and flexibility of the vertebral column [5]. The healthy intervertebral disc consists of the inner nucleus pulposus (NP) encompassing the annulus fibrosus (AF) which, together with the cartilaginous endplates, abut the vertebrae [6]. The nucleus is rich with proteoglycan, an incompressible shock absorber which under compressive loads, distributes hydraulic pressure in all directions [3,6], while the AF consists of collagen type I, elastin and fibroblast-like cells. The IVD is aneural and avascular, receiving nutrition from adjacent cartilaginous endplates [3]. However, due to aging, as well as various environmental factors (e.g., mechanical stress and osmotic pressure), the IVD is susceptible to mechanical and chemical degeneration. At the molecular level, the IVDH is mostly related to the extracellular matrix molecules and collagen degradation, resulting in the upregulation of their degradation products as well as the upregulation of pro-inflammatory cytokines [7,8]. Once the degenerative process starts, a biochemical and mechanical cascade of events is triggered that can ultimately lead to structural failure of the IVD and clinical signs of diseases [5,9].

IVDH is a common cause of spinal cord injury (SCI) in dogs, resulting in the compression of the spinal cord, associated with pain as well as sensory and motor deficits. The severity of neurological signs is determined by neuroanatomical location, velocity and the amount of the compressive material as well as the duration of the compression [10]. Primary SCI refers to the initial mechanical insult, whereas secondary injury is a biochemical cascade following the primary event and consists of vascular dysregulation, neurogenic shock, oxidative stress and excitotoxicity, ischemia and inflammatory cascade [4]. Nevertheless, the IVDH-related pathophysiological processes remain poorly understood, especially at the molecular level. Diagnosis at an early stage of the disease could help to restore disc structure and mechanical structure by using personalized and targeted therapeutic strategies, circumventing the painful symptoms in patients [11,12].

The use of state-of-the-art technology and instrumentation has enabled the identification and quantification of metabolites and large biomolecules in complex matrices with high precision, reproducibility and sensitivity, using low analyte concentrations. Shotgun tandem mass tag (TMT)-based quantitative proteomics enables high-throughput multiplex analysis on up to sixteen samples simultaneously. Unlike proteomics, metabolomics is a powerful tool to reveal metabolomic panels since metabolites are common intermediate and end products of complex biochemical cascades linking the genome, transcriptome and proteome to the phenotype [13]. High-resolution mass spectrometry-based proteomics and metabolomics, combined with the application of various bioinformatic tools and pipelines, have been widely used for studying the pathophysiological processes in central nervous system (CNS) diseases and other neurological conditions by analyzing tissues and biological fluids such as serum or cerebrospinal fluid (CSF) [13,14,15]. Although not easily accessible, unlike blood, due to its multiple functions such as structural, hydrodynamic, metabolic and immunological, CSF is the only readily accessible biofluid that evaluates current CNS status and is a key consideration in differential diagnostic procedures in neurodegenerative disorders [14,15,16]. The major constituent of cerebrospinal fluid is water (99%), whereas the remaining 1% are ions, proteins, glucose, lactose and other organic compounds and cells. The chemical composition of CSF is affected by CNS-related pathophysiological processes [15]. 

Both human and animal clinical studies are mainly focused on a specific omics layer rather than applying a multi-omics approach, which is the main reason why full omics integration is still lacking, especially those providing statistical methods for consistent multi-omics data handling [16]. However, the number of integromics-related studies is constantly increasing. The integration of multi-omics data through data- and knowledge-based approaches enables insight into complex events occurring at the molecular level that are being reflected in various pathophysiological processes in organisms and that cannot be observed by studying just one component of a biological system. Finally, the major challenge in multiple omics data integration lies in the selection of appropriate tools for a given research question [17]. 

In our study, integrative MS-based high-resolution proteomic and untargeted metabolomics analyses of CSF have been employed to gain deeper insights into changes occurring during IVDH in dogs, monitoring the pathophysiological processes at the protein and metabolite levels. Furthermore, since there is no “golden standard” for integrating proteomic and metabolomics data, we designed a classification model to identify the most important features and, by using data-mining tools, reveal significant pathways affected by this degenerative disease. Special focus was placed on identifying the most consistent features being the revelation of potential biomarkers of spinal cord compression, as well as the effect of changes in CSF on CNS and vital functions. The understanding of the patient’s unique profiles at the molecular level and pathways affected could help to improve the diagnosis of this disease and select specific treatments to provide the best outcome.

## 2. Results

### 2.1. Proteomic Analysis and Proteomic Data Validation

Proteomic analysis of CSF was performed using a gel-free tandem mass tag label-based proteomic approach without the depletion of high abundant proteins. Prior to the analysis, total protein concentration in CSF was measured. However, there were no significant differences in total protein concentration between the control and IVDH groups (data not shown). Data-dependent analysis (DDA) enabled the identification of 626 CSF proteins according to set criteria (two unique peptides, 5% false discovery rate, FDR), among which 204 proteins were master proteins. The information about peptide spectrum matches (PSMs), including identified peptides, proteins and protein groups is available in Supplementary File S1. The principal component analysis (PCA) score plot revealed two clear clusters of samples, with the IVDH group being more heterogeneous based on TMT-based proteomic results (Supplementary Figure S1). 

**Figure 1:**
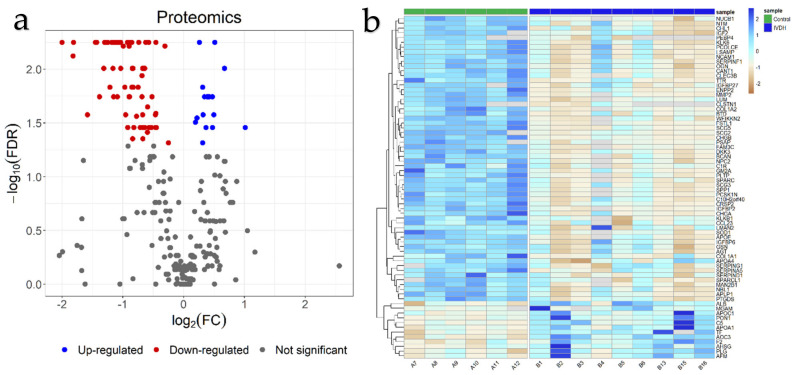
Statistical analysis of proteomics results: (**a**) Volcano plot of evaluated groups after false discovery rate (FDR) correction. Proteins overexpressed in intervertebral disc herniation (IVDH) group are in red, lower-expressed are in blue. (**b**) Heatmap revealing the differentially expressed genes in the control and intervertebral disc herniation groups. Each row represents the fold change (FC) expressed as log2FC of a single gene across all samples. Figure 1b is also available within Supplementary Files for detailed insight (Supplementary Figure S9).

In total, 227 proteins were statistically significantly differential in abundance due to the disc herniation after applying FDR correction (Supplementary File S2, Figure 1a,b), with a majority of proteins (89%) being down-regulated in the IVDH group compared to the control group. Of those, 73 proteins were determined as being related to unique genes (Supplementary Table S1) with the ten most upregulated and downregulated proteins shown in Table 1. 

According to gene ontology analysis (FDR corrected *p*-values < 0.05) using the Database for Annotation, Visualization and Integrated Discovery (DAVID) tools, proteins with significant differences in abundances within the control and IVDH group were involved in calcium ion binding (30.6%), serine-type endopeptidase inhibitor activity (19.4%), heparin binding (13.8%), cholesterol binding (11.1%), phosphatidylcholine-sterol O-acyltransferase activator activity (8.3%), collagen binding (8.3%) and fatty acid binding (8.3%), as can be found in Supplementary Figure S2 and Supplementary File S3. 

**Figure 2:**
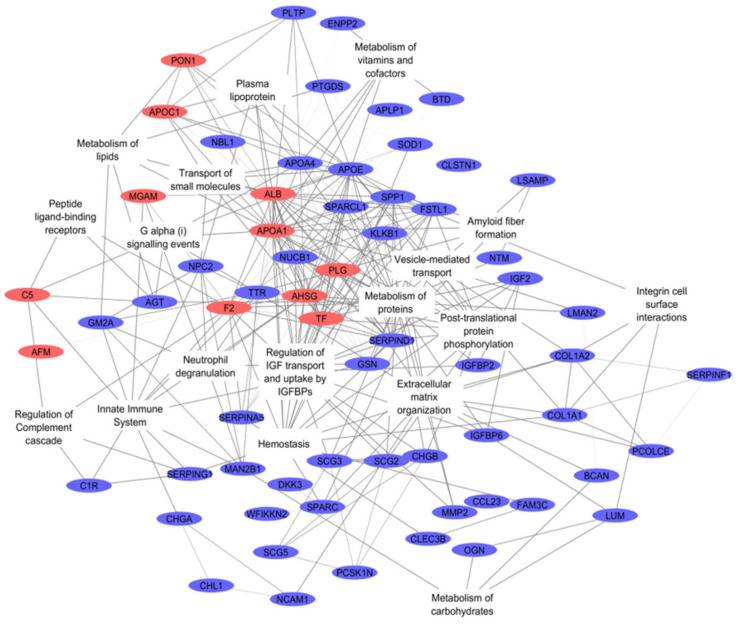
Interactome of pathways differentially expressed between control and intervertebral disc herniation (IVDH) groups and their intermediate proteins. The network was visualized using the Cytoscape using the results of REACTOME pathway analysis and protein-protein interactions identified with STRINGdb. Proteins overexpressed in IVDH are in red, lower-expressed are in blue.

REACTOME pathway analysis (Supplementary File S3; filtered list presented in Table 2) revealed that most of the CSF proteins differing in abundance are involved in the metabolism of proteins (26, *p* = 3.46 × 10^−8^), the immune system (19, *p* = 2.70 × 10^−4^), hemostasis (19, *p* = 1.14 × 10^−11^) and the regulation of Insulin-like Growth Factor (IGF) transport and uptake (19, *p* = 5.86 × 10^−23^). The major interacting proteins involved in the most significant pathways are depicted in Figure 2.

**Figure 3:**
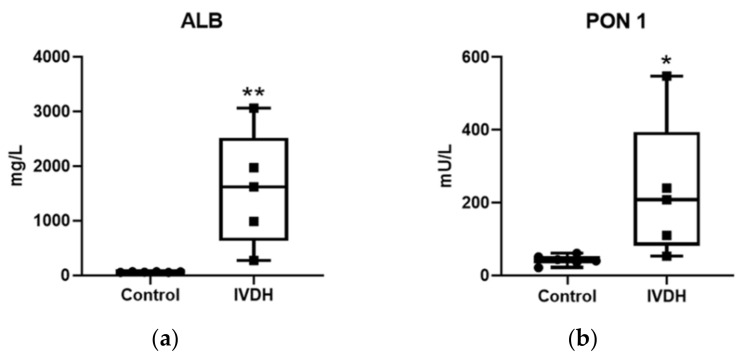
Analytical validation of proteomic results: (**a**) measured activity of paraoxonase 1 (PON1), (**b**) albumin (ALB) concentration in cerebrospinal fluid of intervertebral disc herniation (IVDH) group compared to the control group. * *p* < 0.05 (*p* = 0.0375 herein), ** *p* < 0.01 (*p* = 0.0058 herein).

The differences in protein abundance revealed by MS-based proteomic analysis were evaluated by measuring the activity of paraoxonase 1 (PON1) as well as albumin concentration in CSF samples to validate the proteomic results. Significant changes in the abundance of albumin (difference between means ±SEM = 1522.0 ± 423.4; *p* = 0.0058) and PON1 activity (difference between means ±SEM = 189.6 ± 77.79 mU/L; *p* = 0.0375) were found in the IVDH group (Figure 3). The obtained results for albumin and PON1 are in line with the proteomic findings; however, the validation results (if converted to fold change) have higher fold changes than those obtained by proteomic analysis, which cloud be explained by the sensitivity of TMT technology [18].

### 2.2. Metabolomics

**Figure 4:**
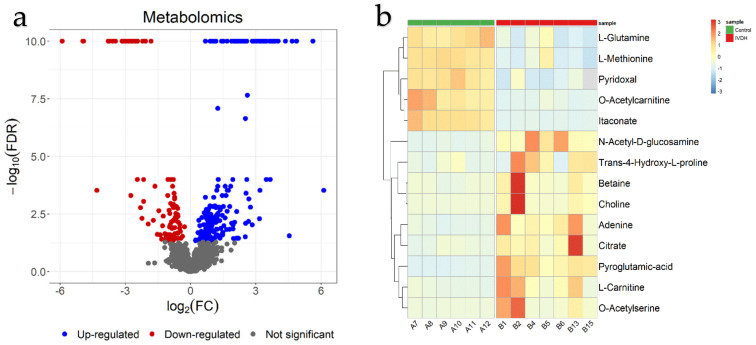
Statistical analysis of proteomics results: (**a**) Volcano plot of evaluated groups after false discovery rate (FDR) correction. Proteins overexpressed in intervertebral disc herniation (IVDH) group are in red, lower-expressed are in blue.; (**b**) Heatmap revealing the differentially expressed genes in the control and IVDH groups. Each row represents fold change (FC) expressed as the log2FC of a single gene across all samples.

Global metabolome analysis using the zwitterionic-polymeric hydrophilic interaction chromatographic separation of metabolites in CSF resulted in 2941 peaks representing metabolic features, which were annotated by database search or identified on the basis of mass and mass/retention time match to known standards, respectively. Among them, 55 unique compounds were matched to a known standard and another 3798 potential metabolites were annotated in (Polyomics integrated Metabolomics Pipeline) PiMP, using available databases. The list of metabolites generated using PiMP software is available in Supplementary File S4. A PCA score plot revealed that the metabolomic profiles of the studied groups (healthy control and IVDH) could be clearly distinguished (Supplementary Figure S3) and 291 metabolic features referring to 73 metabolites according to PiMP analysis (Figure 4a, Supplementary File S4) were significantly changed. Additionally, 14 significantly changed metabolites were identified using standards (Table 3, Figure 4b). Total ion chromatograms showing CSF metabolites for both groups in positive and negative ion mode are shown in Supplementary Figures S4–S7. 

KEGG Pathway analysis performed in PiMP revealed the CSF metabolites changed due to the IVDH are involved in C5-branched dibasic acid metabolism (*p* = 7.6 × 10^−9^), D-glutamine and D-glutamate metabolism (*p* = 7.5 × 10^−6^), and Arginine and proline metabolism (*p* = 2.8 × 10^−4^), among others, as reported in Table 4.

### 2.3. Integration of Omics Data

**Figure 5:**
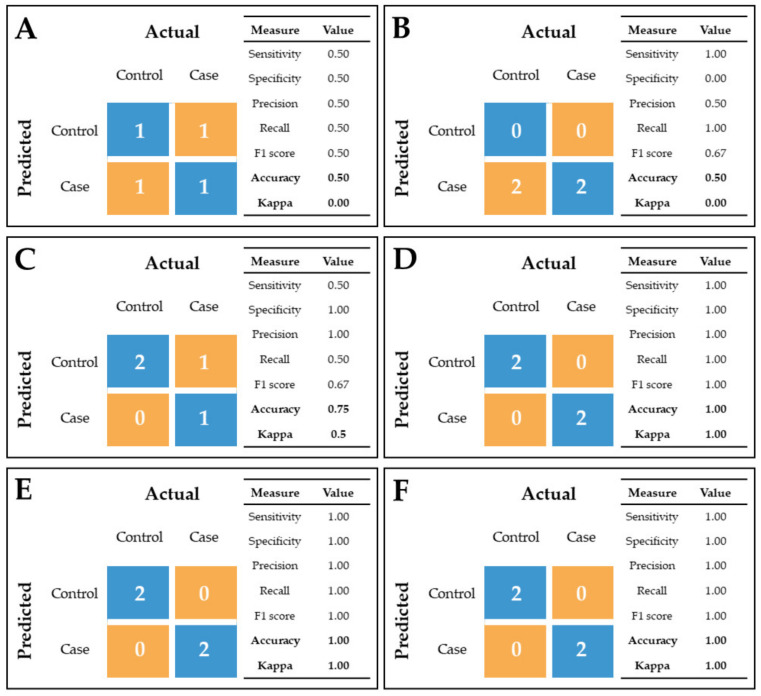
Performance of different classification models on test data using different algorithms and options for feature selection in the form of classification matrices: (**A**) minimal-redundancy-maximal-relevance (mRMR)–10 top-ranked features; (**B**) mRMR–20 top-ranked features; (**C**) mRMR–30 top-ranked features, (**D**) recursive feature elimination (RFE)–7 features selected; (**E**) RFE–9 features selected; (**F**) Combined mRMR (30 top-ranked features) and RFE (7 and 9 features selected)–only features in overlap in 2/3 models were used (6 features).

Subgroups of significantly differentially abundant protein and metabolite sets were subsequently analyzed by applying both data-driven and knowledge based-approaches as specified within the methods section. Firstly, in order to extract the important information from the obtained omics features, PCA analysis was performed, showing the separation of samples when plotted against the first two principal components (Supplementary Figure S8). Furthermore, we built various classification models with integrated data from 16 significant and identified metabolites and 73 significant and unique proteins. In addition, we used two different algorithms for feature selection—recursive feature elimination (RFE) and minimal-redundancy-maximal-relevance (mRMR) algorithm, and the results (in the form of confusion matrices and feature ranks based on their importance) are reported in Figure 5. The RFE algorithm showed two best models with 7 and 9 features selected, while the best performance of mRMR algorithm showed one model with the top 30 ranked features. Since these classification models are built upon a very small training set (only 9 samples), in order to have some samples left for the test set (4 samples), we built a final classification model from the features that were constantly selected, regardless of the algorithm. Thus, features appearing in 2/3 models (RFE with 7 and 9 selected features and mRMR with top 30 ranked features) were used for the final classification model. Selected features for the final classification model were: follistatin Like 1 (FSTL1), secretogranin V (SCG5), nucleobindin 1 (NUCB1), calcitonin receptor-stimulating peptide 2 precursor (CRSP2), N-acetyl-D-glucosamine and adenine. The final model, as well as models that used the RFE algorithm, correctly classified all the samples in the test set.

**Figure 6:**
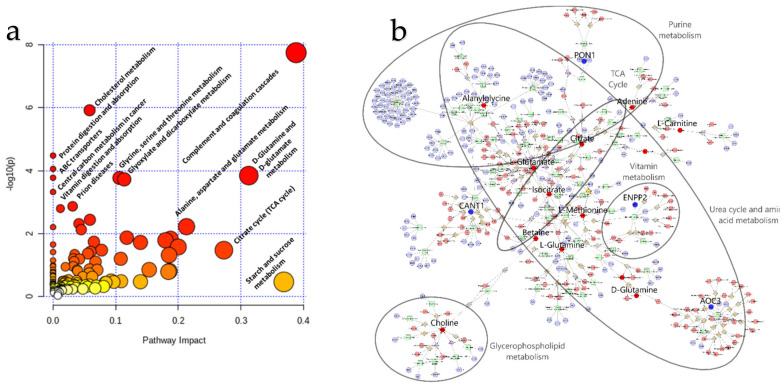
(**a**) Joint pathway enrichment analysis performed in MetaboAnalyst reveals the key pathways in cerebrospinal fluid affected by intervertebral disc herniation (IVDH) in dogs. (**b**) Pathway-based Compound-Reaction-Enzyme-Gene (C-R-E-G) network obtained using significantly deregulated genes and metabolites visualized in Cytoscape plugin Metscape (nods: blue—gene, red—compound/metabolite, green—enzyme). Highlighted blue (gene) and red (metabolite) nods represent input data. Enlarged feature names, together with the metabolic pathways, represent the features closely related to the IVDH. The C-R-E-G network is also available as a Supplementary File for better insight into various features (Supplementary Figure S9).

Furthermore, to obtain the biological significance of our omics data, dual (metabolite and protein) pathway analysis was performed using all significant proteomic and metabolomic features. Both joint pathways that can be observed in the CSF due to IVDH are shown in Table 5, Figure 6a and Supplementary File S5. Furthermore, the pathway-based network of significant features was also visualized by Metascape (Figure 6b).

## 3. Discussion

Spine-related disorders represent significant health concerns for both humans and dogs, affecting the quality of life. Histologic, biomechanical, compositional and clinical similarities have enabled comparative studies between these species, making the canine IVDH model tremendously useful for both understanding the pathophysiology of disease and validating novel therapies prior to clinical trials [19]. The degenerative disc changes are commonly diagnosed by magnetic resonance imaging (MRI), X-ray or computed tomography (CT) scans [20]. Furthermore, total protein concentration elevation in CSF partially reflects the functional status of the nerve root (e.g., nerve root compression), leaking of plasma proteins through the blood–nerve root barrier into the CSF or can be indicative of a tumor [21,22]. However, the total protein concentration difference is mostly not significant in the CSF of IVDH dogs, in line with our previous research [22]. Finally, the pathogenesis involves multiple molecular events that cannot be identified by applying the above-mentioned methods and requires a more advanced systems approach. Understanding those molecular events may provide a better understanding of imbalances which could lead to more effective personalized treatments [20,23]. To obtain an insight into the complex biological processes in IVDH occurring at the molecular level, we used the novel integrative multi-omics approach, involving the integration of high-resolution mass spectrometry-based proteomics and global metabolome profiling data, which has not previously been applied in IVDH in human or veterinary medicine in IVDH. In relation to this current topic, and according to the best authors’ knowledge, IVDH-related proteomic studies have been published to address quantitative analyses of IVD [24], secretome and proteoglycan in canine NP [25], as well as gel-based approaches in human CSF in IVDH [26]. Although there are recent papers reporting multi-omics (e.g., proteomics, metabolomics, transcriptomics and genomics) studies contributing to the identification of potential biomarkers in neurovascular diseases [27], the use of biostatistical omics data integration is still at an early stage [28]. 

In our study, the LC-MS/MS analysis of TMT-labelled CSF tryptic peptides enabled the identification of 73 master proteins involved in calcium binding, lipid, vitamin and amino acid metabolisms as well as immune response, presenting the potential protein biomarker candidates of IVDH. This information, although valuable for further potential clinical diagnostic application, requires further studies and clinical validation. Accordingly, it can be expected that some proteins, with regards to their abundance in the CSF, are showing limited biological intra-individual variations in the CSF due to age and gender (proteins that originate from blood, serotransferrin, vitamin D-binding protein, ganglioside GM2 activator, beta-2-glycoprotein) unlike inflammatory proteins and proteins specific to CNS in humans [29]. Based on the PCA plot (Supplementary Figure S1), it can be observed that global proteomic profiles of selected individual animals within groups are not so consistent; however, they are clearly separated from the control group. It can be concluded accordingly that the changes in abundance are at their highest extent caused by the disease. 

MS results presented herein were analytically validated by quantitative measurements of albumin concentration and enzyme activity of PON1 in the CSF of IVDH dogs compared to the control group, where the CSF of five dogs with IVDH (not previously used for omics analyses) and the CSF of six healthy dogs were used. The proteins used for validation were selected based on both biological importance and test availability. Albumin is the major protein in CSF, exclusively produced by the liver; thus, its concentration in CSF depends on the blood–brain barrier (BBB). If total protein concentration in the CSF increases, albumin concentration increases disproportionally due to molecular size and blood–brain barrier (BBB) disruption. Since albumin was found to be abundant in the CSF of human patients following incomplete spinal cord injury at 1–8 days post-injury, being a potential biomarker for spinal cord injury progression and recovery [30], it was selected for analytical validation. In our study, elevated concentration of albumin in the CSF of IVDH group revealed by TMT label-based proteomics was also confirmed by using immunoturbidimetric assay (Figure 3). The antioxidant PON1 has an anti-inflammatory role and was documented in the serum, CSF, and brain, suggesting its activity in neurodegenerative disease. Furthermore, PON1 plays an important role in keeping the homeostatic balance of intervertebral disc and for this reason, it was selected for analytical validation. Unlike in our results, CSF PON1 activity was reported to decrease with the progression of neurodegenerative diseases and Alzheimer’s disease, as well as dementia [18]. However, plasma PON1 activity in animal models of spinal cord injury increases [31] and the cases studied in this investigation were more related to this type of injury rather than degeneration and lead to an increase in PON1. Increased levels of PON1 and its activity can be explained by PON1 having a protective role in the early phase of oxidative stress because of antioxidant enzyme consumption [31]. The increase in CSF PON1 activity was also obtained by assay, using p-nitrophenyl acetate as a substrate herein, confirming the proteomic findings.

The CSF metabolomics profile mainly reflects brain metabolic processes but also consists of intermediate and end products of neurotransmission and energy metabolism. Among others, metabolic changes in CSF can be caused by various pathophysiological processes, such as inflammation or response to oxidative stress responses [32], which was also the case in IVDH dogs in our study. To fill the existing gaps in IVDH-related knowledge, it is necessary to obtain detailed insight into the metabolomic profile. We are aware that due to metabolite diversity, it is impossible to cover all metabolites by a single method and/or using only one analytical platform, and this is probably the reason why some, for example, lipid metabolites related to lipid peroxidation, neuroinflammation and changes in antioxidant compounds like glutathione [32,33] were not identified/detected as changed in the CSF herein. However, although known IVDH-related important metabolites might be absent, pathway analysis revealed that C5-Branched dibasic acid metabolism, D-Glutamine and D-glutamate metabolism, Arginine and proline metabolism, Glutathione metabolism and Nitrogen metabolism are significantly affected by IVDH. All above-mentioned pathways were reported significantly affected in spinal cord damage, including demyelination in multiple sclerosis [33]. Branched-chain amino acids are crucial for brain metabolism and function as they are an important source of pyruvate in energy metabolism and serve as nitrogen donors in protein synthesis [34]. Arginine metabolism, nitrogen metabolism and glutathione metabolism are linked to oxidative stress. Arginine serves as a precursor to nitric oxide and changes within arginine metabolism might result in the synthesis of nitric oxide. The formation of reactive nitrogen species is cytotoxic for nerves, promoting myelin destruction [34]. Although we did not find the glutathione itself to be aberrant, as a protective compound against oxidative stress by oxidizing to glutathione disulfide (GSSG) with a simultaneous reduction of H2O2, the concentration alliteration of metabolites such as pyroglutamic acid [35] revealed the deregulation of glutathione metabolism in IVDH. The elevation of pyroglutamic acid has not yet been related to IVDH, unlike anion-gap metabolic acidosis [36] or cancer [37], according to the authors’ knowledge. Affected metabolic pathways and the alliteration of antioxidant defense molecules such as an increase in superoxide dismutase type 1 (SOD1) imply that oxidative stress may also play an important role in IVDH. IVDH-affected pathways in the CSF also included D-glutamine and D-glutamate metabolism, and glutamate was also found to be significantly elevated in IVDH dogs. Glutamate is the major excitatory neurotransmitter in the CNS and its extracellular accumulation points out the abnormalities in the glutamatergic neurotransmission [38]. Elevated concentrations of glutamate have catastrophic impacts on nervous tissue due to the overstimulation of glutamate receptors, which can lead to subsequent excitotoxic injury of glial cells and neurons [39]. The increase of glutamate in the CSF has been related to several neurological and brain pathologies such as multiple sclerosis, Alzheimer’s and Parkinson’s disease or during ischemia [39].

### 3.1. Integromics Model Reveals Key Molecules for IVDH

The main task of omics data integration, besides obtaining knowledge about disease using various omics layers, is to be able to straightforwardly classify patients with a high specificity based on the best omics feature selection. For our pilot study, the challenge was to use a well-defined (as can be seen from the omics PCA plots, Figure 1 and Figure 3) but small population size with multiple proteomic and metabolomic features for the selection of the most suitable data-driven and data-mining tools in order to define the pathways and the CSF features best representing the IVDH in dogs that can be used for disease diagnosis, monitoring and/or progression. Data-driven statistical methods integrate multi-omics data sets performing sample clustering or classification, equalizing the contribution of different omics data which could ignore biological relationships between different types of molecules [17]. Since better performance can be achieved by combining several selection algorithms [40], we used RFE and mRMR feature selection algorithms to integrate the analysis of multi-omics data addressing three major challenges [41], specifically the effect of dimensionality, the differences in scales, and extracting the most relevant features across different multi-omics results. mRMR and RFE algorithms have been applied widely in omics analyses of gene expression data for glioblastoma prognosis [42] and cancer classification [43]. 

In our study, the six most significant omics features selected by the optimal classification model were FSTL1, SCG5, NUCB1, CRSP2, N-acetyl-D-glucosamine and adenine, respectively. However, their biological role should be determined by data mining. Data mining computational tools derive pathways and molecular interactions from the correlation structures, which provides the results with the biological significance [17]. However, biological knowledge (molecular interactions and pathway data) is under permanent change by data curators due to new data generation and is thus biased, with the density of connections in certain regions of the network affected by (in)completeness of experimentally proven molecular interactions and their availability within databases [42]. To determine the effectiveness of our classification model, data mining of biological significance and the feature’s potential relation to IVDH revealed FSTL1, SCG and CRSP2 as key molecules in studies addressing neuropathic pain [44]. Furthermore, NUCB1 was reported as a novel pan-neuronal calcium handling marker, correlated to Alzheimer’s disease [45]. Unlike those, N-acetyl-D-glucosamine has a neuroprotective role as a regulator of primary myelination and myelin repair and is of importance in demyelinating diseases like multiple sclerosis [46]. Finally, adenine is, as one of the purines, involved in neuromodulation, neurotransmission, activation of differentiation and neuritogenesis of precursor cells and neurons. Additionally, purines also contribute to immunological response due to astrocytes and microglia activation, initiating the inflammatory reactions [46]. The diagnostic significance and potential are yet to be examined by further studies.

### 3.2. Integrative Omics Analysis Reveals Key Processes Referring to IVDH

**Figure 7:**
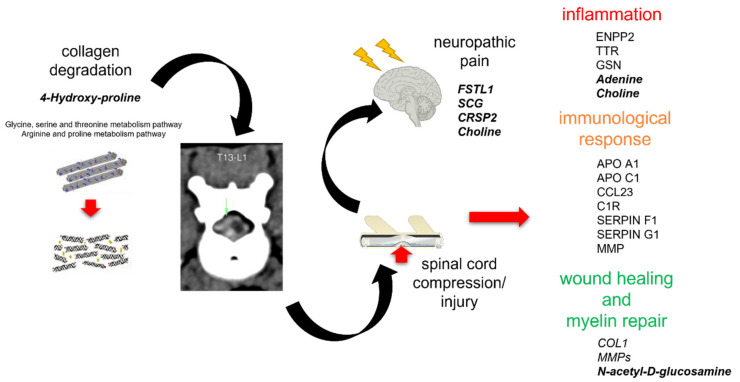
Complex processes occurring in cerebrospinal fluid of dogs with intervertebral disc herniation (IVDH) revealed by integrative omics approach pointing out the key molecules (in bold) proposed by our classification data-driven model. Related proteins: follistatin Like 1 (FSTL1), calcitonin receptor-stimulating peptide 2 precursor (CRSP2), secretogranin (SCG), nucleobindin 1 (NUCB1), Ectonucleotide Pyrophosphatase/Phosphodiesterase 2 (ENPP2), transthyretin (TTR), gelsolin (GSN), apolipoproteins (APO A1 and APO C1), C-C Motif Chemokine Ligand 23 (CCL23), Complement C1r (C1R), Serpins (SERPIN F1 and G1), matrix metalloproteinases (MMP) and collagen 1 (COL1).

Although not easily accessible as serum or saliva due to technical and ethical reasons (especially in the case of healthy individuals), CSF was proven to be a very informative biofluid for omics analyses. Herein, by using an integromics approach of proteomic and metabolomic data, we demonstrated that a plethora of events occurs in IVDH in dogs, that can be observed by CSF analyses at the molecular level (Figure 7). Multiple features were found, revealing the most common anomaly—a manifestation of degenerative changes in terms of collagen (COL1) degradation, causing IVDH, which may result in nerve damage, spinal cord injury (such as deficiency of apolipoprotein E (APO E)) [47] and neuropathic pain (such as a higher representation of mimecan and/or apolipoprotein C1) [48].

Annulus fibrosus, the outer layer of intervertebral disc, is, together with elastin, fibroblast-like cells and proteoglycans composed of collagen type I [3]. Collagen is a biological polymer continually synthesized and degraded in the extracellular space. However, among dogs with IVDH, excessive collagenolysis has been demonstrated in diseases such as atherosclerosis, arthritis and even cancer. Collagen is composed of tropocollagen, containing repeating triplets of amino acids X1, X2 and glycine (X1–X2–G), with many X1/X2 occupied by either proline or hydroxyproline [49]. Integrated omics and metabolomic analyses emphasized the glycine, serine and threonine metabolism pathways as significantly affected by IVDH, with 17 annotated and 7 identified metabolites (45% coverage, as shown in Table 3), as well as the arginine and proline metabolism pathways having 31 annotated and 7 identified metabolites (42% coverage, as shown in Table 3). In our study, 4-hydroxyproline was found to be elevated in the CSF of the IVDH group, showing collagen degradation [50]. Following annotation based on molecular ion *m/z* and fragment ions (Supplementary File S4), other collagen fragments presented as series metabolites isoleucyl-proline, glycyl-hydroxyproline and valyl-glycine, and were also found to be different in concentration in the IVDH group (however, not significantly according to our results). Unlike previous metabolomic studies of collagen degradation-related diseases in nerve, serum and/or urine, other collagen degradation products such as amino acids (glycine, proline, alanine, leucine and valine) were not found to be significantly changed in this study, which might be related to the analytical method/platform selectivity or degree of disc degradation [50,51,52]. Interestingly, the statistically lower abundance of collagen (COL1) was also found in the CSF of dogs in the IVDH group, which might be explained by collagen deposition during wound healing [53].

When collagen degrades, the fibrous ring ruptures and the protrusion of the nucleus pulposus can press on a nerve root and/or the spinal cord, bursting the nucleus of the disc and damaging the nerve and compressing the spinal cord. Interestingly, unlike altered proteins related to spinal cord injury (transferrin–TF, matrix metalloproteinases–MMPs, insulin-like growth factor 1–IGF-1 and apolipoprotein E–APO E) [54,55], no metabolites reported in both untargeted and targeted metabolomics SCI-related studies [56] were found to be altered. This might be explained by the severity of SCI itself in the IVDH group, which was mild. However, we found evidence of wound-healing events such as myelination and myelin repair (N-acetyl-D-glucosamine, MMPs), angiogenesis and neurogenesis (MMPs). Due to spinal cord compression and nerve injury, inflammation and/or ischemia occur, which are important processes of disc herniation. Changes in inflammation markers were associated with proteins such as APO A1, C-C Motif Chemokine Ligand 23 (CCL23) and MMP2. APO A1 is the principal protein fraction of high-density lipoproteins (HDL), involved in reverse cholesterol traffic, as well as inflammatory and immune response. It has a protective role against cardiovascular diseases and neuroinflammation in neurodegenerative diseases [57]. Furthermore, CCL23 was found to be deranged in the CSF in our study. Unlike some other studies addressing neuroinflammation where it was increased, albeit not significantly [58], neuroinflammation was in contrast found to be decreased in our study. CCL23 is a chemokine—a circulating and tissue-inflammatory molecule, being a chemotactic factor for monocytes/macrophages, dendritic cells and lymphocytes, up-regulating proinflammatory cytokine release. CCL23 was proposed to be a blood biomarker for the early diagnosis of cerebral damage [59]. CCL23 is expressed in response to IL-4 and LPS, TNFα and IFN-γ and signals through CCR1 [60]. In our case, the patients had acute IVDH, and the enzymatic cascade enabling the release of CCL23 was probably not yet activated. Matrix metalloproteinases (MMPs) are involved in the pathogenesis of neuroinflammatory diseases [61]. MMP2 (72-kDa gelatinase A) can be found in a healthy brain, in neurons from the cortex, cerebellum and CSF. It was found to be decreased in the CSF of the IVDH group, as reported in neuroinflammation [62]. Additionally, MMP2 specifically cleaves type IV collagen, the major structural component of basement membranes, increasing the permeability of BBB. MMP-2 is also important in remyelination, angiogenesis and neurogenesis [62]. Inflammation processes caused by cell injury/tissue damage initiate both innate and adaptive immune responses, which was also determined in our study [63]. Multiple proteins already documented as being involved in the maintenance of CNS and immune surveillance against injured cells have been altered in IVDH dogs; for instance, complement C1R, whose activity is tightly regulated to protect host cells, and its regulator SERPIN G1 (which binds to and inactivates C1r, C1s and MASP-1/2 proteases) [64]. Lower levels of serpin protein C1 inhibitor activate kinin pathways and increase levels of bradykinin in the blood, which is responsible for capillary leakage and angioedema [65]. Cell injury (tissue damage), nerve root compression and swelling, as well as disc inflammation, may have an important role in pain generation. The bioinformatic analysis of CSF proteins related to neuropathic pain gathered from multiple studies [66] identified three groups of proteins involved in inflammatory responses (APO A1, APO C1 [49], SERPIN F1), immune responses (ectonucleotide pyrophosphatase/phosphodiesterase 2 (ENPP2), transthyretin (TTR), gelsolin (GSN)) and metabolic processes (albumin, prothrombin (F2)), and these were all found changed in our study. The application of metabolomics in pain research is still at an early stage [67]. Notwithstanding this issue, the metabolomics of the CSF in pain patients revealed that neuropathic pain alters sphingomyelin–ceramide metabolism, nervous tissue damage (alanine, taurine) and inflammatory processes (choline) [67,68,69]. Choline, which was found to be elevated in IVDH dogs in our study, is a metabolite related to glial activity, and its elevation was found to be linked with worse pain interference [69].

Our pilot study using canine clinical samples was constrained by sample size restrictions (especially in the control group due to animal ethical guidelines), as well as CSF sample volume availability, testing research hypotheses in a small number of subjects. Furthermore, ideal matching based on canine age and the weight of the control group compared to the study group was not possible for operational reasons (especially related to owners’ consent for CSF collection in healthy dogs and ethical guidelines). However, our findings are considered to be a valid indication of the change in CSF due to intervertebral disc herniation.

## 4. Materials and Methods

### 4.1. Experimental Design 

A total of 14 client-owned dogs presented to the veterinary clinics of the Faculty of Veterinary Medicine, University of Zagreb, Croatia, as well as 6 shelter dogs were involved in this pilot study. The sample size was determined based on recommendations for statistical consideration of optimal clinical study design in proteomics in preliminary studies for biomarker discovery [70,71], taking into consideration gender, age, sample type, quantification method (isobaric labeling) and availability of samples due to ethical restrictions (it is ethically not acceptable to collect CSF in healthy dogs for the purpose of scientific research, only because the procedure of sampling requires general anesthesia and cerebellomedullary puncture, which is an invasive method). The current pilot study was provided to patients who were treated at the Veterinary Faculty, University of Zagreb. The owners signed a written consent for participation in the study on the acceptance of the risk of anesthesia and surgical procedure. All dogs were submitted for a general physical examination, and general hematology was performed. Prior to general anesthesia, pre-anesthetic assessment of patients was performed. 

The omics studies involved canine patients classified into two groups. Healthy dogs (control group) (N = 6) were mixed breeds (20 kg average body weight), age 2–6 years, while the intervertebral disc herniation group (N = 9) were mixed breeds (body weight 6–15 kg), age 6–12 years. In IVDH dogs, disc herniation was detected in T13/L1/L2 discs by CT. The dogs in IVDH group were paraplegic with preserved deep pain reflex and underwent a surgical procedure for decompression of the spinal cord. Additionally, 5 samples from IVDH group not used for omics studies were randomly selected for the analytical validation (age 6–12 years). The Ethical Committee at the Faculty of Veterinary Medicine, University of Zagreb, Croatia approved the research (No. 640-01/18-17/67; Record No. 251-61-21/333-18-01). 

### 4.2. Sample Collection

Cerebrospinal fluid was sampled under general anesthesia from cisterna magna prior to operation. Induction to general anesthesia was obtained with propofol (Propofol®, Abbott, Maidenhead, UK), administered intravenously and maintained with sevoflurane (Sevorane®, Abbott, Maidenhead, UK) applied through an endotracheal tube. Anesthesia was adjusted to minimally interfere with cerebral blood flow and intracranial pressure [72]. The CSF was collected into sterile Eppendorf tubes (size 1.5 mL), immediately centrifuged at 3000× *g* at 4 °C for 5 min, aliquoted into Eppendorf tubes (size 1.5 mL) to avoid multiple freeze-thaw cycles and stored at −80 °C till analyzed. All samples were collected within 6 months. 

### 4.3. Proteomic Analysis

Proteomic analysis of CSF samples (IVDH N = 9 and control N = 6) was performed by using a gel-free TMT-based quantitative approach, as described previously [18]. In short, total protein concentration was determined using a bicinchoninic acid assay (BCA assay) (Thermo Scientific, Rockford, IL, USA). An internal standard was prepared by mixing equal protein amounts of each CSF sample. An amount of 35 µg CSF proteins was diluted to a volume of 100 µL using 0.1 M triethylammonium bicarbonate (TEAB, Thermo Scientific, Rockford, IL, USA), reduced (5 µL of 200 mM dithiothreitol for 60 min, 55 °C) (Sigma Aldrich, St. Louis, MO, USA), alkylated (5 µL of 375 mM IAA at 30 min, room temperature in the dark) (Sigma Aldrich, St. Lois, MO, USA) and acetone-precipitated (600 µL, overnight, −20 °C) (Merck, Darmstadt, Germany). The obtained protein pellets were dissolved in 50 µL of 0.1 M TEAB and digested using trypsin gold (1 mg/mL, Promega; trypsin-to-protein ratio 1:35, at 37 °C overnight). For labeling, TMT tenplex reagents (Thermo Scientific, Rockford, IL, USA) were prepared as recommended by manufacturers and 19 µL of each reagent were used for labeling. The internal standard was labeled with TMT *m*/*z* 131, while other samples were randomized within two TMT experiments. Labeling reaction was quenched by 5% hydroxylamine (Sigma-Aldrich, St. Louis, MO, USA). Per experiment, nine TMT-labelled peptide samples were combined with the internal standard, aliquoted, dried and stored at −80 °C for subsequent LC-MS/MS analysis. 

High-resolution LC-MS/MS analysis was performed using an Ultimate 3000 RSLCnano system (Dionex, Germering, Germany) coupled with a Q Exactive Plus mass spectrometer (Thermo Fisher Scientific, Bremen, Germany) as reported elsewhere [18]. Differentially TMT-labelled peptides were desalted on the trap column and separated on the analytical column (PepMap™ RSLC C18, 50 cm × 75 μm, Thermo Scientific, Rockford, IL, USA) using linear-gradient 5–45% mobile phase B (0.1% formic acid in 80% ACN) over 120 min, at the flow rate of 300 nL/min. Mobile phase A consisted of 0.1% formic acid in LC-MS-grade water. Ionization was achieved using a nanospray Flex ion source (Thermo Fisher Scientific, Bremen, Germany) equipped with a 10 μm-inner diameter SilicaTip emitter (New Objective, Littleton, MA, USA). The MS operated in positive ion mode using DDA Top8 method. Full scan MS spectra were acquired in range from *m*/*z* 350.0 to *m*/*z* 1800.0 with a resolution of 70,000, 120 ms injection time, AGC target 1E6, a ± 2.0 Da isolation window and the dynamic exclusion 30 s. For HCD fragmentation, step collision energy (29% and 35% NCE) with a resolution of 17,500 and AGC target of 2E5 were used. Precursor ions with charge states of +1 and more than +7, as well as unassigned charge states, were excluded from fragmentation. 

For protein identification and reporter ion-based relative quantification, the SEQUEST algorithm implemented into Proteome Discoverer (version 2.3., Thermo Fisher Scientific, Bremen, Germany) was applied. Database search against *Canis lupus familiaris* FASTA files (downloaded from NCBI database 04/04/2019, 172,083 entries) was performed using parameters as follows: two trypsin missed-cleavage sites, precursor and fragment mass tolerances of 10 ppm and 0.02 Da, respectively; carbamidomethyl (C) fixed peptide modification, oxidation (M) and TMT sixplex (K, peptide N-terminus) dynamic modifications. The false discovery rate (FDR) for peptide identification was calculated automatically based on decoy database by the Percolator algorithm within the Proteome Discoverer workflow and was set at 1%. Proteins containing at least two unique peptides and 5% FDR were reported as identified. The mass spectrometry proteomics data were deposited to the Consortium via the PRIDE partner [73] repository with the dataset identifier PXD024921. 

### 4.4. Analytical Validation of Proteomic Results

Frozen aliquots of CSF (IVDH N = 5 and control N = 6) were thawed to determine paraoxonase 1 (PON1) activity, as well as albumin (ALB) concentration using automated biochemistry analyzer Abbott Architect c4000 (Abbott, Chicago, IL, USA). PON1 activity was assayed using the method of Tvarijonaviciute *et al*. with p-nitrophenyl acetate (Sigma-Aldrich, Saint Louis, MO, USA) as substrate [74]. Albumin concentration in CSF samples was evaluated with human immunoturbidimetric assay (Microalbumin OSR6167 Beckman Coulter, Brea, CA, USA) which was previously validated for use in canine CSF samples [75]. 

### 4.5. Untargeted Metabolomic Analysis

Metabolomics analysis of CSF samples (IVDH N = 7 and control N = 6) was performed using an untargeted approach, as described [76]. In short, pooled sample was prepared by mixing 10 µL of each sample (control and IVDH). Matrix blank sample contained extraction solvent. For metabolite extraction, a volume of 25 µL of each sample was subjected to chloroform/methanol/water (1:3:1, *v*/*v*/*v*) extraction on a vortex mixer for two hours at 4 °C. All samples (CSF samples, pooled sample, matrix blank) were subsequently centrifuged (13,000× *g* for 5 min at 4 °C). The supernatant (200 µL) was stored at −80 °C until LC-MS/MS analysis. 

Metabolite extracts were separated on a 4.6 mm × 150 mm zwitterionic-polymeric hydrophilic interaction chromatography (ZIC-pHILIC) column (Merck SeQuant, Darmstad, Germany) using Dionex UltiMate 3000 RSLC system (Dionex, Germering, Germany) coupled to a Thermo Orbitrap Q Exactive Plus (Thermo Fisher Scientific, Hemel Hempstead, UK) using a gradient of 80% to 95% of mobile phase A for 15 min at 25 °C and a flow rate of 0.3 mL/min, as reported [76]. Mobile phase A contained 20 mM ammonium carbonate in water and B contained 100% acetonitrile. The injection volume was 10 μL. Samples were maintained in the autosampler at 5 °C prior to injection. The mass spectrometer was operated by altering positive and negative modes with electrospray ionization at a resolution of 70,000 and the full scan *m*/*z* range of 70–1050. The MS settings for positive electrospray ionization were used as follows: source voltage of 3.8 kV, sheath gas 40 (arbitrary units), auxiliary gas of 5 (arbitrary units) and capillary temperature of 320 °C. A standard mix containing a mix of 148 authentic compounds—metabolites—was used for metabolite identification. A standard mix (containing reference compounds for metabolite identification) and quality control samples used in metabolomics analysis herein were kindly donated by Dr. Richard Burchmore, Glasgow Polyomics, College of Medical, Veterinary and Life Sciences, University of Glasgow, UK. Metabolomics data were deposited to the EMBL-EBI MetaboLights [77] database (DOI: 10.1093/nar/gkz1019, PMID:31691833) with the identifier MTBLS2689. 

Metabolomics data were analyzed using the Polyomics integrated Metabolomics Pipeline (PiMP), available at http://polyomics.mvls.gla.ac.uk (accessed on 14 January 2021), using standard workflow and default parameters [78]. In short, raw data was converted from the ‘RAW’ file format (Thermo Scientific) to an open-source ‘mzXML’ file format, centroided and split into positive and negative polarities using MSConvert tool [79]. Metabolite identification was performed in PiMP, following Metabolite Standards Initiative guidelines by matching retention times and accurate masses of detected peaks with either the authentic standards or were annotated (assigned putatively based on accurate masses) using metabolite libraries search (e.g., The Human Metabolome Database, HMDB, and/or Kyoto Encyclopedia of Genes and Genomes, KEGG) integrated within PiMP standard workflow. The metabolic maps provided within PiMP software were derived from the KEGG database. 

### 4.6. Statistical and Bioinformatic Analyses

#### 4.6.1. Proteomics

Proteins that had more than 50% missing values were excluded from the subsequent statistical analysis. Statistics were performed using R v3.2.2 [80]. Sample outliers were detected per each group for each protein using Dixon’s test from R package outliers v0.14 [81]. Significant outliers (*p* < 0.05) were removed from further analysis. To test the difference in protein abundance between groups, Mann–Whitney U test was performed. Fold change (FC) between two groups was calculated as mean(IVDH)/mean(control) and expressed on log2 scale. PCA and volcano plots were designed using R package ggplot2 v3.1.1 [82]. 

For bioinformatics, proteins GI accession numbers were converted into official gene symbol by DAVID conversion tool (https://david.ncifcrf.gov/conversion.jsp, accessed on 19 March 2021) and UniProtKB ID mapping (https://www.uniprot.org/uploadlists/, accessed on 19 March 2021). Protein–protein interactions and REACTOME pathway analysis was performed using STRINGdb v11.0 (https://string-db.org/, accessed on 26 March 2021) [83], with the selection of Canis lupus familiaris default settings with the exception of no more than 5 interactors to show in the 1st shell. Networks of relationship between REACTOME pathway and proteins with significantly differential abundances between groups were designed using Cytoscape v.3.7.1. (https://cytoscape.org/, accessed on 10 June 2021) [84]. Annotation terms (GO Biological process, GO Molecular function, GO Cellular component) were generated using Database for Annotation, Visualization and Integrated Discovery (DAVID) v6.7. (https://david.ncifcrf.gov/, accessed on 10 June 2021) [85].

#### 4.6.2. Proteomic Data Validation

The significance of PON1 activity within CSF samples of healthy and IVDH dogs, as well as albumin concentrations in CSF, was determined by applying the Mann–Whitney U test within GraphPad Prism software (v.8.4.2.). 

#### 4.6.3. Metabolomics

Metabolomics raw data were submitted to the PiMP for metabolite annotation/identification, statistical analysis and metabolic pathway analysis using a standard workflow [78]. One group-wise comparison was undertaken to identify differences between control and IVDH group (fold changes expressed as log2FC). Peaks with an (Benjamini–Hochberg) adjusted *p*-value less than 0.05 were considered significant. Further analyses and visualization of PiMP-exported data (peak intensities, log2FC and FDR values of identified/annotated metabolites) were performed using R v.3.2.2. (volcano plot, PCA plot and heatmap) [80].

#### 4.6.4. Statistical Omics Data Integration

Significantly altered and identified metabolites, as well as significantly altered proteins related to unique genes were merged to build a classification model where only the most associated proteomic and metabolic features would be able to discriminate between healthy dogs and dogs diagnosed with disc herniation. First, samples that have both proteomic and metabolomic data (6 controls and 7 IVDH samples) were randomly divided into training and test set in a 70:30 ratio, leading to 9 samples in the training set and 4 samples in the test set. Metabolic data were first log-transformed for further use. The training set was then scaled, centered and the possible missing values were imputed using K-nearest neighbor imputation. We performed elastic net logistic regression with 5-repeated 10-fold cross-validation to fit the training set. Features were then ranked according to their importance and the model was applied on the test set whose performance was evaluated with a confusion matrix. Due to the multicollinearity issue when dealing with small sample size and large number of integrated proteomic and metabolic features, we applied two feature selection algorithms to reduce the number of features to fit the model. One was recursive feature elimination (RFE) algorithm with 5-repeated 10-fold cross-validation and different numbers of features to retain the most important ones. The second one was minimal-redundancy-maximal-relevance (mRMR) algorithm, which ranks features based on their correlation with the output but removes redundant features. For this, we considered the first 10, 20 and 30 ranked features. R packages caret [86] version 6.0-86, glmnet [87] version 4.0-2 and mRMRe [88] version 2.1.0 were used to build classification models. 

#### 4.6.5. Bioinformatic Omics Data Integration

Joint-pathway analysis of metabolites (compound name with matching fold changes used as input) and proteins (official gene symbol with matching fold changes used as input) that were significantly changed due to IVDH was conducted in MetaboAnalyst v.4.0. by applying *Homo sapiens* (human) as model organism. Further visualization was performed in Cytoscape plugin Metscape [89] v3.1.1. to identify enriched metabolic pathways. For the analysis, the lists of significantly changed genes and metabolites were imported into the Metscape, and human was set as a model organism, to obtain pathway-based Compound-Reaction-Enzyme-Gene (C-R-E-G) network using standard workflow.

## 5. Conclusions

Complex conditions, such as IVDH, require a systems biology-based approach that can reveal not only progressive structural and functional changes of patients but also various pathophysiological processes that could be linked to the disease severity and patient’s outcome. For that reason, we applied proteomic and metabolomic analyses combined with the state-of-the-art bioinformatics tools to obtain insight into the various molecules affected by IVDH. We set up a prediction model to examine the most important CSF features involved in IVDH with diagnostic potential and identified the key molecules, namely proteins FSTL1, SCG5, NUCB1 and CRSP2 and the metabolites N-acetyl-D-glucosamine and adenine, involved in neuropathic pain, myelination, neurotransmission and inflammatory response, respectively. Their clinical application requires further clinical validation and the development of suitable tests for routine analysis. The integromics approach provided new deeper insights into a number of mechanisms and molecular pathways affected in IVDH, which could not be observed or distinguished by exclusively monitoring the individual omics layers, not to mention current diagnostic methods. Our approach and obtained results contribute to the improvement of treatment strategies and personalized care for patients with degenerative spinal disorders.

## Figures and Tables

**Table 1:** The top 10 most significantly changed proteins in cerebrospinal fluid of dogs with intervertebral disc herniation (IVDH) compared to healthy dogs. Complete list of all significantly changed proteins can be found in Supplementary File S2.

Higher in Abundance in IVDH Group (Upregulated)				Higher in Abundance in Control Group (Downregulated)			
Accession No. (NCBI)	Adjusted *p*-Value	log2FC	Gene Symbol	Accession No. (NCBI)	Adjusted *p*-Value	log2FC	Gene Symbol
359321961	0.0147	0.31	F2	345789648	0.0056	−2.00	CHGB
73975389	0.0349	0.37	AFM	1418342647	0.0075	−1.82	SCG2
1239925760	0.0181	0.39	C5	345795204	0.0056	−1.81	SCG5
18139619	0.0181	0.41	PLG	3046976	0.0266	−1.58	COL1A1
1418515495	0.0181	0.43	AHSG	1239931864	0.0056	−1.43	ENPP2
2147092	0.0181	0.48	ALB	73970011	0.0181	−1.38	C10H2orf40
3915607	0.0266	0.50	APOA1	1418314244	0.0098	−1.31	TTR
1239918269	0.0056	0.52	AOC3	1239976444	0.0056	−1.30	FSTL1
73975797	0.0098	0.68	PON1	1418222978	0.0056	−1.30	CRSP2
1418304654	0.0349	1.01	APOC1	197085524	0.0056	−1.30	CRSP4

**Table 2:** REACTOME pathways affected by intervertebral disc herniation in canine cerebrospinal fluid (filtered list based on the relation to the disease). Complete list can be found in Supplementary File S3.

REACTOME Pathway Name	ID	# Genes	# Background Genes	Adjusted *p*-Value
Regulation of Insulin-like Growth Factor (IGF) transport and uptake by Insulin-like Growth Factor Binding Proteins (IGFBPs)	HSA-381426	19	123	5.86 × 10^−23^
Post-translational protein phosphorylation	HSA-8957275	13	106	2.79 × 10^−14^
Platelet activation, signaling and aggregation	HSA-76002	16	256	1.04 × 10^−13^
Hemostasis	HSA-109582	19	601	1.14 × 10^−11^
Extracellular matrix organization	HSA-1474244	12	298	2.69 × 10^−8^
Metabolism of proteins	HSA-392499	26	1948	3.46 × 10^−8^
Binding and uptake of ligands by scavenger receptors	HSA-2173782	6	40	3.17 × 10^−7^
Intrinsic pathway of fibrin clot formation	HSA-140837	5	22	7.83 × 10^−7^
ECM proteoglycans	HSA-3000178	6	75	7.48 × 10^−6^
Degradation of the extracellular matrix	HSA-1474228	7	139	1.30 × 10^−5^
Post-translational protein modification	HSA-597592	17	1366	6.26 × 10^−5^
Plasma lipoprotein remodeling	HSA-8963899	4	30	7.28 × 10^−5^
Chylomicron remodeling	HSA-8963901	3	10	1.20 × 10^−4^
Innate immune system	HSA-168249	14	1012	1.20 × 10^−4^
Regulation of complement cascade	HSA-977606	4	47	2.70 × 10^−4^
Neutrophil degranulation	HSA-6798695	9	471	3.70 × 10^−4^
Crosslinking of collagen fibrils	HSA-2243919	3	18	3.90 × 10^−4^
Metabolism of vitamins and cofactors	HSA-196854	6	185	4.20 × 10^−4^
Amyloid fiber formation	HSA-977225	4	78	1.30 × 10^−3^
Integrin cell surface interactions	HSA-216083	4	83	1.60 × 10^−3^
Activation of matrix metalloproteinases	HSA-1592389	3	33	1.60 × 10^−3^
Diseases associated with glycosaminoglycan metabolism	HSA-3560782	3	40	2.50 × 10^−3^
Collagen degradation	HSA-1442490	3	64	7.20 × 10^−3^
Glycosphingolipid metabolism	HSA-1660662	2	44	3.68 × 10^−2^
Glycosaminoglycan metabolism	HSA-1630316	3	122	3.22 × 10^−2^

**Table 3:** List of identified (using authentic standards) significantly changed metabolites in cerebrospinal fluid of intervertebral disc herniation (IVDH) group compared to healthy dogs obtained using the Polyomics integrated Metabolomics Pipeline (PiMP).

Higher in Abundance in IVDH Group (Upregulated)				Higher in Abundance in Control Group (Downregulated)			
Peak ID	Metabolite	log2FC	Adjusted *p*-Value	Peak ID	Metabolite	log2FC	Adjusted *p*-Value
693	Hydroxyproline	0.76	3.00 × 10^−2^	2325	Itaconate	−3.78	5.1 × 10^−10^
23	Betaine	1.08	4.16 × 10^−2^	858	O-Acetylcarnitine	−2.73	4.6 × 10^−6^
1789	Citrate	1.19	1.44 × 10^−2^	847	Pyridoxal	−2.30	0.0017
1410	N-Acetyl-D-glucosamine	1.46	5.00 × 10^−4^	2717	L-Methionine	−2.16	0.0009
688	Adenine	1.51	1.50 × 10^−2^	2070	L-Glutamine	−0.83	0.0002
217	L-Carnitine	1.65	1.51 × 10^−2^				
24	Choline	1.79	1.46 × 10^−2^				
466	L-Glutamate	2.51	3.10 × 10^−2^				
245	Pyroglutamic acid	2.52	2.30 × 10^−7^				

**Table 4:** Metabolic pathways affected by intervertebral disc herniation in canine cerebrospinal fluid generated by the Polyomics integrated Metabolomics Pipeline (PiMP) using KEGG Pathway.

KEGG Pathway Name	Pathway ID	Assigned Formulas	Total Pathway Formulas	Pathway Coverage (%)
C5-Branched dibasic acid metabolism	99	7	21	33.33
D-Glutamine and D-glutamate metabolism	55	4	7	57.14
Glyoxylate and dicarboxylate metabolism	93	11	48	22.92
Ascorbate and aldarate metabolism	7	12	25	48.00
Arginine and proline metabolism	35	25	79	31.65
GABAergic synapse	218	3	9	33.33
Histidine metabolism	37	21	41	51.22
Butanoate metabolism	98	6	30	20.00
Pentose and glucuronate interconversions	4	8	23	34.78
Chlorocyclohexane and chlorobenzene degradation	41	3	57	5.26
Citrate cycle (TCA cycle)	2	4	15	26.67
Phosphotransferase system (PTS)	155	3	25	12.00
Cysteine and methionine metabolism	28	11	52	21.15
Glutathione metabolism	58	5	29	17.24
Proximal tubule bicarbonate reclamation	243	5	16	31.25
Glutamatergic synapse	215	2	7	28.57
Nitrogen metabolism	124	2	17	11.76
Vitamin B6 metabolism	106	4	29	13.79
Glycerolipid metabolism	73	4	14	28.57
ABC transporters	152	32	80	40.00
Alanine, aspartate and glutamate metabolism	24	9	23	39.13
Purine metabolism	20	10	78	12.82
Glycerophospholipid metabolism	76	3	23	13.04
HIF-1 signaling pathway	167	3	12	25.00
Zeatin biosynthesis	122	4	30	13.33
Bile secretion	251	10	89	11.24
Pyrimidine metabolism	23	13	56	23.21
Galactose metabolism	6	5	21	23.81
Mineral absorption	253	13	26	50.00

**Table 5:** KEGG pathways affected by intervertebral disc herniation in dogs observed in cerebrospinal fluid as obtained using MetaboAnalyst taking into consideration significantly deregulated proteins and metabolites.

KEGG Pathway Name	Total Features in Pathway	*p*/FDR *	Impact *
Complement and coagulation cascades	80	5.83 × 10^−2^	0.3881
Cholesterol metabolism	60	1.99 × 10^−4^	0.0583
D-Glutamine and D-glutamate metabolism	18	7.86 × 10^−3^	0.3125
Glycine, serine and threonine metabolism	90	7.86 × 10^−3^	0.1059
Glyoxylate and dicarboxylate metabolism	92	7.86 × 10^−3^	0.1136
Prion diseases	38	4.54 × 10^−2^	0.0313
Mineral absorption	87	4.87 × 10^−2^	0.0118

* The *p*-value after false discovery rate (FDR) correction was calculated during the pathway enrichment analysis in while the pathway impact value was calculated from the pathway topology analysis in MetaboAnalyst.

## Data Availability

The mass spectrometry proteomics data have been deposited to the Consortium via the PRIDE partner repository with the dataset identifier PXD024921. Metabolomics data have been deposited to the EMBL-EBI MetaboLights database (DOI: 10.1093/nar/gkz1019, PMID:31691833) with the identifier MTBLS2689. All results are presented within the manuscript and/or Supplementary Materials.

## Supplementary Materials

The following are available online at https://www.mdpi.com/article/10.3390/ijms222111678/s1, Supplementary File S1. List of PSMs, peptides, proteins, master proteins and protein groups identified in CSF of healthy dogs and dogs with IVDH obtained by TMT-based proteomics; Supplementary File S2. Differentially abundant proteins in CSF of healthy dogs and dogs with IVDH: Supplementary File S3. Gene ontology and pathway analyses of differentially abundant proteins in CSF of healthy dogs and dogs with IVDH; Supplementary File S4. Metabolomics profiling of CSF of healthy dogs and dogs with IVDH revealing peaks, identified and annotated metabolites, metabolic pathways and metabolic maps; Supplementary File S5. Joint pathway analysis of differentially abundant CSF proteins and metabolites generated in MetaboAnalyst reveals the pathways affected by IVDH in dogs.
