# Supplementary material for: Multi-Omics Approach to Elucidate Cerebrospinal Fluid Changes in Dogs with Intervertebral Disc Herniation
Source: Int J Mol Sci. 2021 Oct 28;22(21):11678. doi: 10.3390/ijms222111678 (PMC8583948; doi:10.3390/ijms222111678)
Supplement: Supplementary file 1 [file ijms-22-11678-s001.zip › Supplementary data.pdf]

Supplementary data

Multi-omics approaches for elucidating cerebrospinal fluid changes in dogs with intervertebral disc herniation

Anita Horvatić[1*], Andrea Gelemanović[2], Boris Pirkić[3], Ozren Smolec[3*], Blanka Beer Ljubić[4], Ivana Rubić[4], Peter David Eckersall[5], Vladimir Mrljak[4], Mark McLaughlin[5], Marko Samardžija[6], Marija Lipar[3]

[1] Department of Chemistry and Biochemistry, Faculty of Food technology and Biotechnology, University of Zagreb, Zagreb, Croatia

[2] Mediterranean Institute for Life Sciences, Split, Croatia

[3] Clinic for Surgery, Orthopaedics and Ophthalmology, Faculty of Veterinary Medicine, University of Zagreb, Zagreb, Croatia

[4] Internal Diseases Clinic, Faculty of Veterinary Medicine, University of Zagreb, Zagreb, Croatia

[5] Institute of Biodiversity, Animal Health & Comparative Medicine, College of Medicine, Veterinary Medicine and Life Sciences, University of Glasgow, Glasgow, UK

[6] Reproduction and Obstetrics Clinic, Faculty of Veterinary Medicine, University of Zagreb, Zagreb, Croatia

*To whom correspondence should be addressed:

Anita Horvatić, ahorvatic@pbf.hr; Ozren Smolec, osmolec@vef.hr

LIST OF SUPPLEMENTARY FILES:

Supplementary file S1. List of PSMs, peptides, proteins, master proteins and protein groups identified in CSF of healthy dogs and dogs with IVDH obtained by TMT-based proteomics

Supplementary file S2. Differentially abundant proteins in CSF of healthy dogs and dogs with IVDH

Supplementary file S3. Gene ontology and pathway analyses of differentially abundant proteins in CSF of healthy dogs and dogs with IVDH

Supplementary file S4. Metabolomics profiling of CSF of healthy dogs and dogs with IVDH revealing peaks, identified and annotated metabolites, metabolic pathways and metabolic maps

Supplementary file S5. Joint pathway analysis of differentially abundant CSF proteins and metabolites generated in MetaboAnalyst reveals the pathways affected by IVDH in dogs

Supplementary Table S1. Statistically significantly differential proteins (unique genes) in abundance due to the disc herniation after applying FDR correction.

Accession	Gene Symbol	Description	FDR	log2FC	Coverage in Percent	Number of Peptides	Number of Unique Peptides	MW in kDa	calc pI	Sum PEP Score	Score Sequest HT
73975389	AFM	afamin	0.0349	0.37	43	24	24	69.1	5.35	113.349	165.85
5821257	AGT	angiotensinogen, partial	0.0266	-0.67	57	9	9	20.2	8.85	101.018	191.56
1418515495	AHSG	alpha-2-HS-glycoprotein	0.0181	0.43	33	9	9	39.2	5.31	129.797	260.43
1418328547	ALB	LOW QUALITY PROTEIN: serum albumin-like	0.0181	0.40	94	79	3	68.6	5.69	1340.786	7624.12
57091057	AOC3	membrane primary amine oxidase isoform X1	0.0181	0.34	6	4	4	84.2	6.37	17.52	27.02
359318692	APLP1	amyloid-like protein 1 isoform X2	0.0061	-0.99	13	6	6	72.5	6.01	30.754	32.45
1418241889	APOA1	apolipoprotein A-I	0.0349	0.47	82	28	28	30.2	5.39	344.37	1031.79
345799905	APOA4	apolipoprotein A-IV	0.0485	-0.25	55	23	23	43.8	5.48	137.638	189.97
1418304654	APOC1	apolipoprotein C-I	0.0349	1.01	22	3	3	9.7	9.41	10.963	22.63
283972739	APOE	apolipoprotein E4, partial	0.0056	-1.10	42	8	8	19.9	10.21	78.697	168.76
548923838	BCAN	brevican core protein precursor	0.0056	-1.16	5	4	4	99.5	4.75	13.728	13.52
545538859	BTD	biotinidase isoform X3	0.0098	-1.03	7	3	3	58.9	6.04	11.946	13.88
73970011	C10H2orf40	augurin	0.0181	-1.38	11	2	2	16.7	8.1	5.152	11.2
1418256182	C1R	complement C1r subcomponent	0.0181	-0.52	24	13	13	80.1	6.24	53.124	59.52
1239925760	C5	complement C5	0.0181	0.39	13	18	18	183.6	6.37	62.701	67.9
1239917098	CANT1	soluble calcium-activated nucleotidase 1 isoform X1	0.0147	-0.85	20	12	12	94.3	6.05	73.366	93.71
1239919521	CCL15	C-C motif chemokine 15 isoform X2	0.0275	-0.77	21	2	2	13.4	8.28	17.35	19.85
57091239	CCL23	C-C motif chemokine 23 isoform X1	0.0275	-0.77	21	2	2	13.5	8.28	17.35	19.85

244539517	CHGA	chromogranin A, partial	0.0147	-1.21	31	13	2	46.9	4.65	77.835	95.6
345789648	CHGB	secretogranin-1 isoform X1	0.0056	-2.00	18	9	9	77.1	5.07	41.095	38.82
1239949328	CHL1	neural cell adhesion molecule L1-like protein isoform X1	0.0098	-0.67	8	6	6	136.7	5.53	22.094	14.2
928162811	CLEC3B	tetranectin	0.0056	-0.92	33	4	4	19.3	6.33	42.913	56.51
1418220357	CLEC3B	tetranectin	0.0056	-0.88	41	6	6	22.2	6.2	60.654	89.73
545499363	CLSTN1	calsyntenin-1 isoform X5	0.0444	-0.67	7	5	5	109.4	4.88	12.489	14.54
3046976	COL1A1	prepro-alpha 1 type I collagen, partial	0.0266	-1.58	16	2	2	18.4	4.48	11.067	6.05
50978940	COL1A2	collagen alpha-2(I) chain precursor	0.0056	-1.06	4	4	4	129.3	9.07	18.812	19.7
1418222978	CRSP2	calcitonin receptor-stimulating peptide 2-like isoform X2	0.0056	-1.30	19	2	2	14.1	5.06	7.853	6.98
50950151	CRSP-2	calcitonin receptor-stimulating peptide 2 precursor	0.0056	-1.30	39	3	3	14.1	5.06	9.661	6.98
197085524	CRSP-4	calcitonin receptor-stimulating peptide 4	0.0056	-1.30	19	2	2	14.2	5.06	7.853	6.98
511093722	DKK3	dickkopf 3 homolog	0.0056	-0.92	41	10	10	37.9	4.59	77.264	81.69
73974395	ENPP2	ectonucleotide pyrophosphatase/phosphodiesterase family member 2 isoform X11	0.0056	-1.45	31	21	21	101.9	7.52	114.272	132.38
359321961	F2	prothrombin	0.0147	0.31	42	19	19	70.3	6	118.26	138.15
1418269977	FAM3C	protein FAM3C	0.0056	-1.10	25	4	4	24.7	8.29	15.024	24.2
1239976444	FSTL1	follistatin-related protein 1	0.0056	-1.30	25	7	7	34.2	5.41	25.312	32.96
359319344	GM2A	ganglioside GM2 activator	0.0056	-1.28	9	2	2	21.6	7.25	8.839	13.21
1239925762	GSN	gelsolin	0.0266	-0.46	31	21	21	88	8.15	157.84	263.73
225216805	IGF2	insulin-like growth factor 2 splice variant 1	0.0115	-0.68	18	3	3	20.2	9.32	11.915	13.97
1239981567	IGFBP2	insulin-like growth factor-binding protein 2 isoform X2	0.0056	-0.62	32	5	5	20.7	7.58	26.148	39.09
1418342184	IGFBP2	insulin-like growth factor-binding protein 2 isoform X1	0.0056	-0.89	27	8	8	35.4	7.21	40.238	53.87
1418328747	IGFBP27	insulin-like growth factor-binding protein 7	0.0181	-0.89	21	6	6	28.9	8.12	22.318	34.55
73996265	IGFBP6	insulin-like growth factor-binding protein 6	0.0349	-0.82	30	5	5	25.3	8.6	41.049	54.68
1418303486	KLK6	kallikrein-6 isoform X1	0.0349	-0.71	36	7	7	32.6	7.65	70.429	91.35

1418255638	KLKB1	plasma kallikrein isoform X1	0.0056	-0.46	23	14	13	70.8	7.96	44.328	57.55
1353234	LMAN2	RecName: Full=Vesicular integral-membrane protein VIP36; AltName: Full=Lectin mannose-binding 2; AltName: Full=Vesicular integral-membrane protein 36; Short=VIP36; Flags: Precursor	0.0349	-0.45	7	2	2	40.2	6.95	4.531	10.73
1418222976	LOC112667675	uncharacterized protein LOC112667675 isoform X1	0.0056	-1.30	9	2	2	28.7	8.65	7.853	6.98
345796043	LSAMP	limbic system-associated membrane protein isoform X5	0.0181	-1.09	8	2	2	37.2	6.98	16.124	14.38
1418336203	LUM	lumican	0.0056	-0.88	34	9	9	38.3	6.68	60.827	100.9
345786397	MAN2B1	lysosomal alpha-mannosidase isoform X2	0.0349	-0.57	7	6	6	113.1	7.66	16.522	19.07
1418219268	MAN2B1	lysosomal alpha-mannosidase isoform X1	0.0098	-0.64	6	5	5	113.2	7.53	13.196	16.37
928156540	MGAM	maltase-glucoamylase, intestinal isoform X2	0.0312	0.20	17	24	24	207.2	5.91	106.311	133.48
7688731	MMP2	matrix metalloproteinase-2, partial	0.0056	-1.26	3	2	2	70.9	5.52	4.648	6.94
1418505711	NBL1	neuroblastoma suppressor of tumorigenicity 1 isoform X1	0.0147	-0.97	10	2	2	19.9	5.07	9.417	15.65
545496657	NCAM1	neural cell adhesion molecule 1 isoform X6	0.0181	-0.69	5	5	5	119	4.81	22.241	24.84
945179	NPC2	CE1	0.0056	-1.17	49	6	6	16	8.02	36.921	50.59
359319464	NTM	neurotrimin isoform X4	0.0181	-0.68	12	3	3	39.1	8	13.355	17.48
1418303986	NUCB1	nucleobindin-1	0.0056	-0.57	7	3	3	53.1	5.11	8.045	8.68
1418302656	OGN	mimecan isoform X1	0.0147	-0.83	22	9	9	42.7	6.62	37.943	58.13
1418307444	PCOLCE	procollagen C-endopeptidase enhancer 1	0.0349	-0.46	27	8	8	48.6	7.09	51.372	53.92
1418248954	PCSK1N	proSAAS	0.0056	-1.28	22	4	4	27.3	6.3	22.427	27.74
1239961997	PEBP4	phosphatidylethanolamine-binding protein 4 isoform X3	0.0444	-0.84	11	2	2	21.5	6.2	5.97	9.27
130314	PLG	RecName: Full=Plasminogen; Contains: RecName: Full=Plasmin heavy chain A; Contains: RecName: Full=Plasmin light chain B	0.0485	0.31	73	15	15	36.7	8.1	126.793	185.32

545540929	PLTP	phospholipid transfer protein	0.0056	-0.74	27	10	10	54.7	7.21	56.487	69.38
73975797	PON1	serum paraoxonase/arylesterase 1	0.0098	0.68	28	8	8	40	5.34	43.225	57.8
73952852	PSAP	prosaposin isoform X3	0.0098	-0.83	19	8	8	58.3	5.03	43.717	50.42
4850328	PTGDS	prostaglandin D synthase	0.0056	-1.09	32	5	5	21.1	7.74	75.535	147.17
1418342647	SCG2	secretogranin-2	0.0075	-1.82	7	4	4	70.7	4.77	12.914	12.52
74000667	SCG3	secretogranin-3 isoform X2	0.0061	-0.90	32	11	11	53.3	5.11	51.737	65.95
345795204	SCG5	neuroendocrine protein 7B2 isoform X1	0.0056	-1.81	12	3	3	23.7	5.58	7.792	12.24
545508405	SERPINA5	plasma serine protease inhibitor	0.0147	-0.75	16	5	5	45.8	8.44	18.586	17.71
345791439	SERPIND1	heparin cofactor 2	0.0061	-0.31	24	11	11	56.6	6.27	48.386	62.66
119637837	SERPINF1	pigment epithelium-derived factor	0.0098	-0.84	47	14	14	44.2	8.68	108.564	158.2
1239944268	SERPING1	plasma protease C1 inhibitor	0.0225	-0.59	26	8	8	44	5.5	68.779	100.36
18150346	SOD1	Cu/Zn superoxide dismutase	0.0056	-1.26	22	2	2	15.9	6.11	10.887	13.43
1418325272	SPARC	SPARC	0.0056	-1.13	25	8	8	34.5	4.78	37.339	41.74
1418201165	SPARCL1	SPARC-like protein 1 isoform X1	0.0098	-1.16	24	11	11	76.2	4.69	91.546	100.12
76161575	SPP1	osteopontin, partial	0.0056	-1.25	18	4	4	29.6	4.56	45.102	62.38
1418510423	TF	serotransferrin-like	0.0056	0.26	76	58	4	78	7.53	819.941	1809.32
1418314244	TTR	transthyretin	0.0098	-1.31	67	7	7	15.9	6.92	52.653	79.15
73966420	WFIKKN2	WAP, Kazal, immunoglobulin, Kunitz and NTR domain-containing protein 2 isoform X1	0.0056	-1.10	13	7	7	63.4	6.4	24.789	24.2

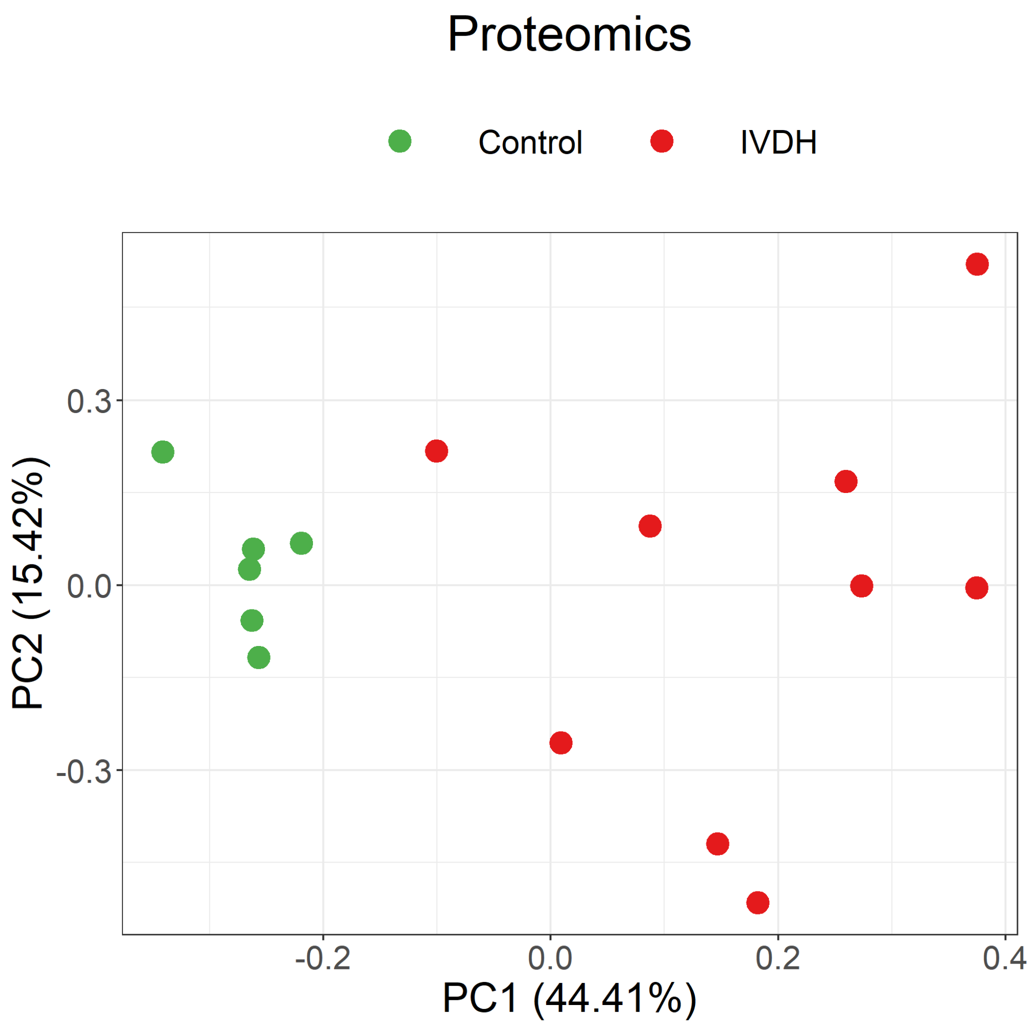

Supplementary Figure S1. Principal component analysis (PCA) plot separates the samples when plotted against the first two principal components based on proteomic data.

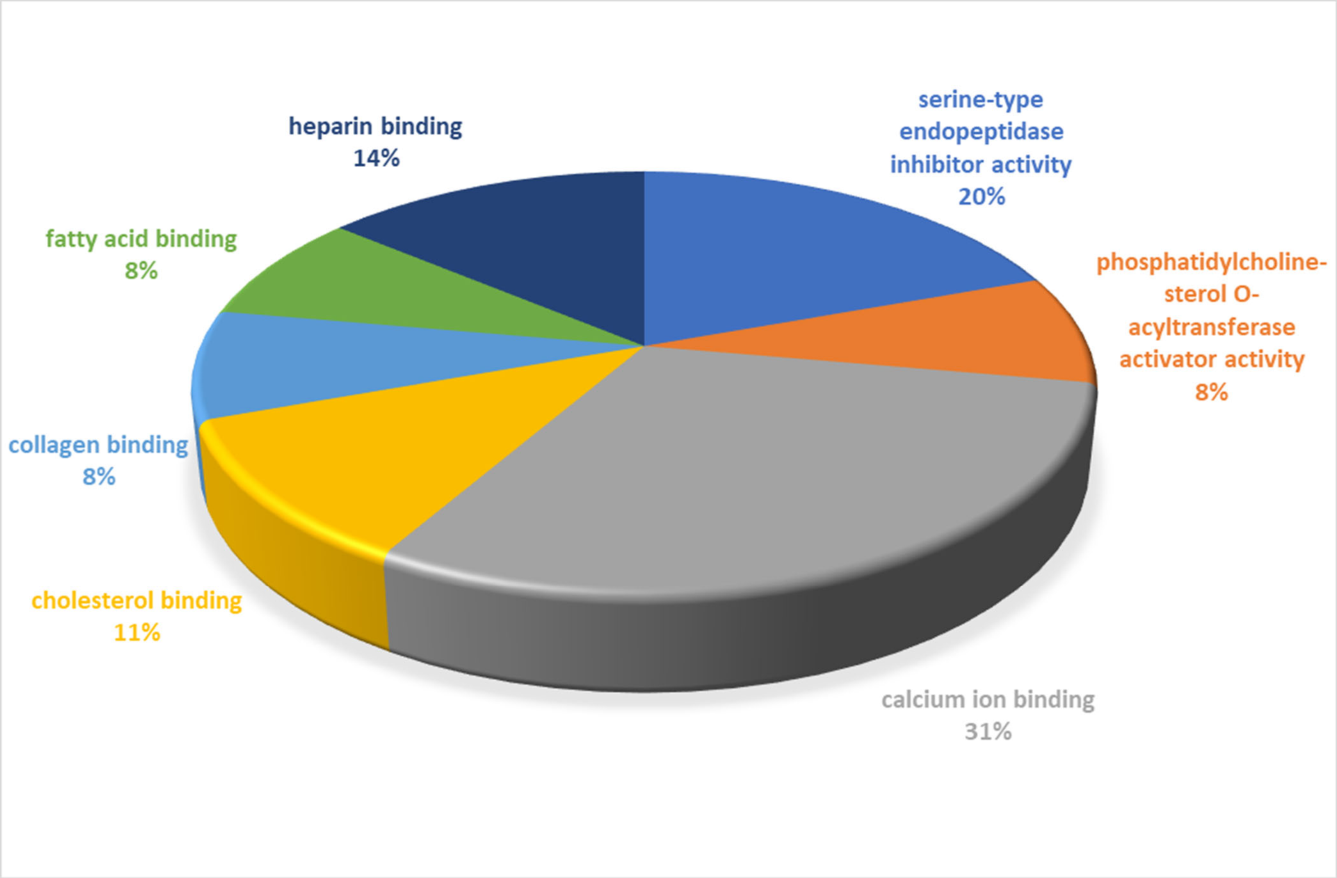

Supplementary Figure S2. Functional classification (molecular function) of proteins with significant differences in abundances within control and IVDH group obtained by DAVID Gene Ontology analysis (FDR corrected P-values < 0.05).

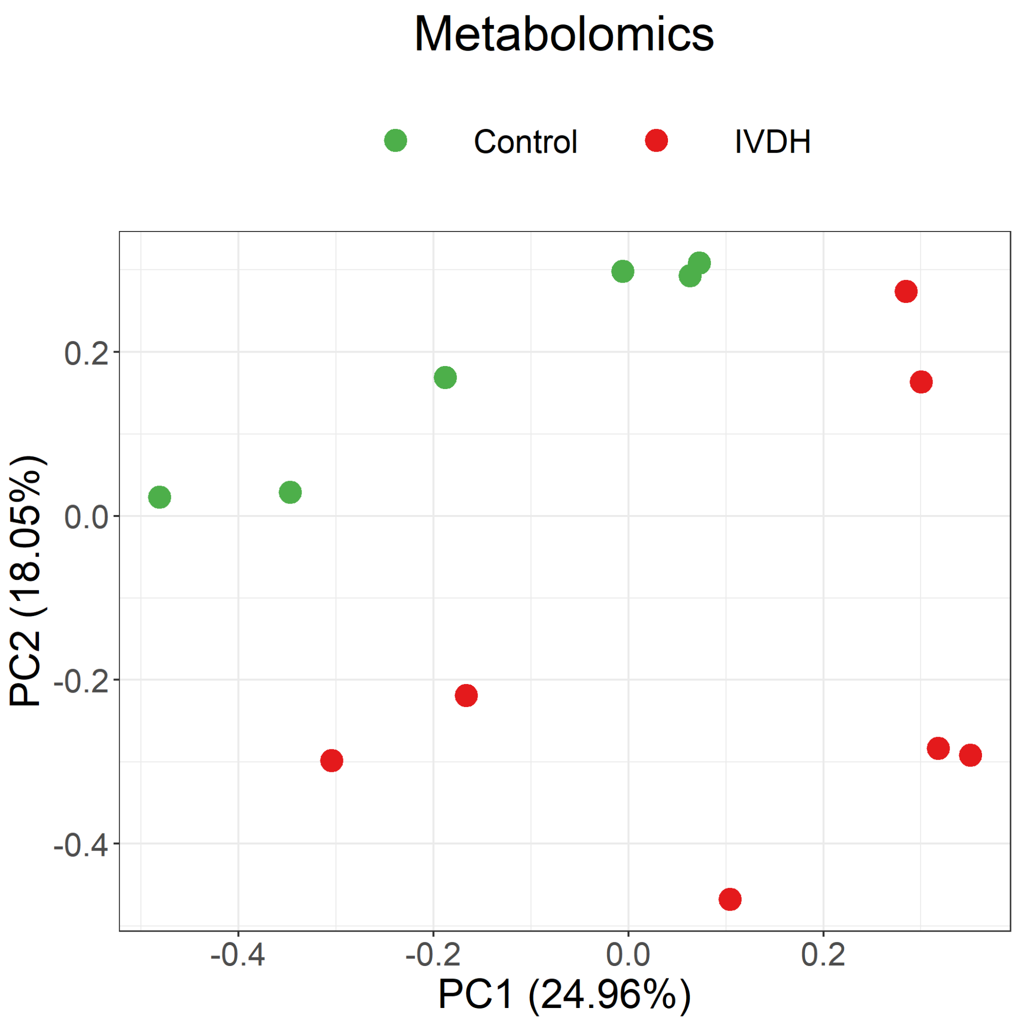

Supplementary Figure S3. Principal component analysis (PCA) of CSF metabolomics features separates the control and IVDH samples when plotted against the first two principal components.

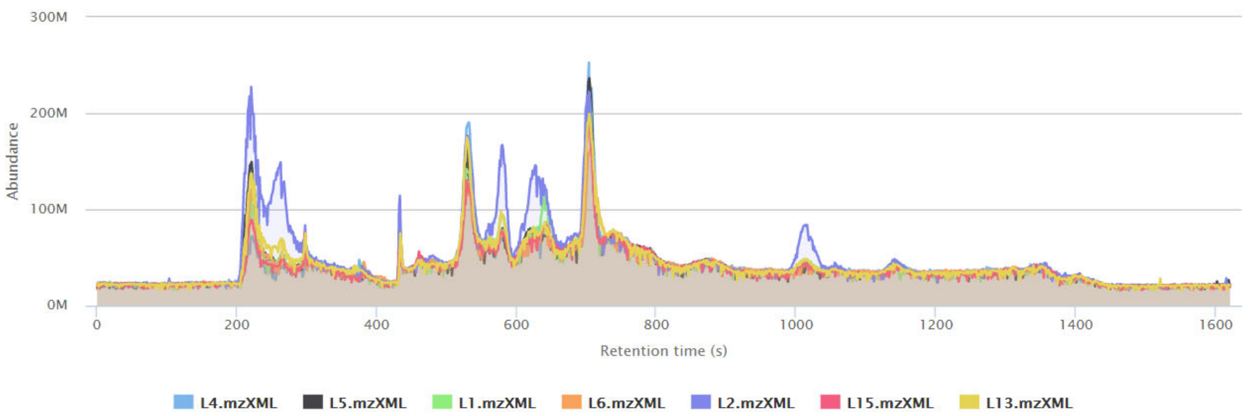

Supplementary Figure S4. Total ion chromatograms of CSF metabolites in IVDH group (positive ion mode) generated in PiMP.

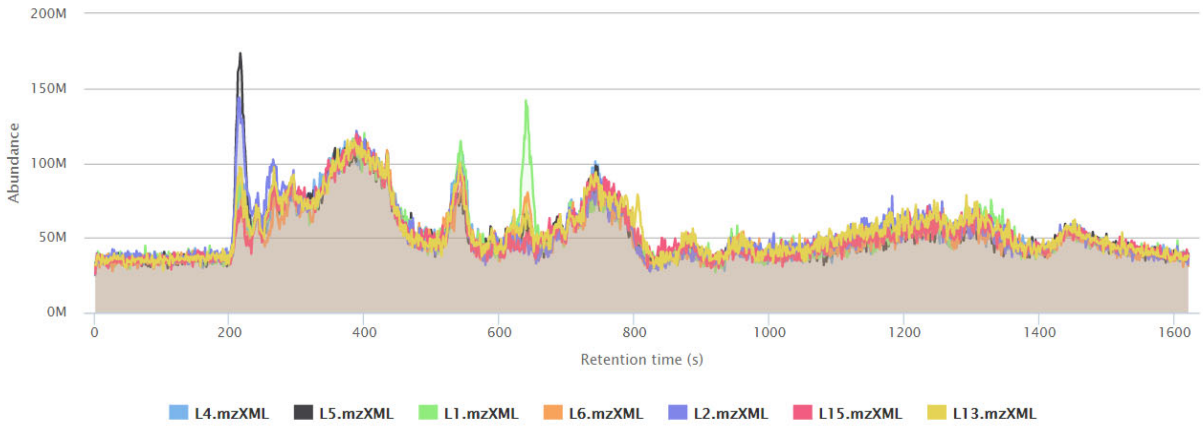

Supplementary Figure S5. Total ion chromatograms of CSF metabolites in IVDH group (negative ion mode) generated in PiMP.

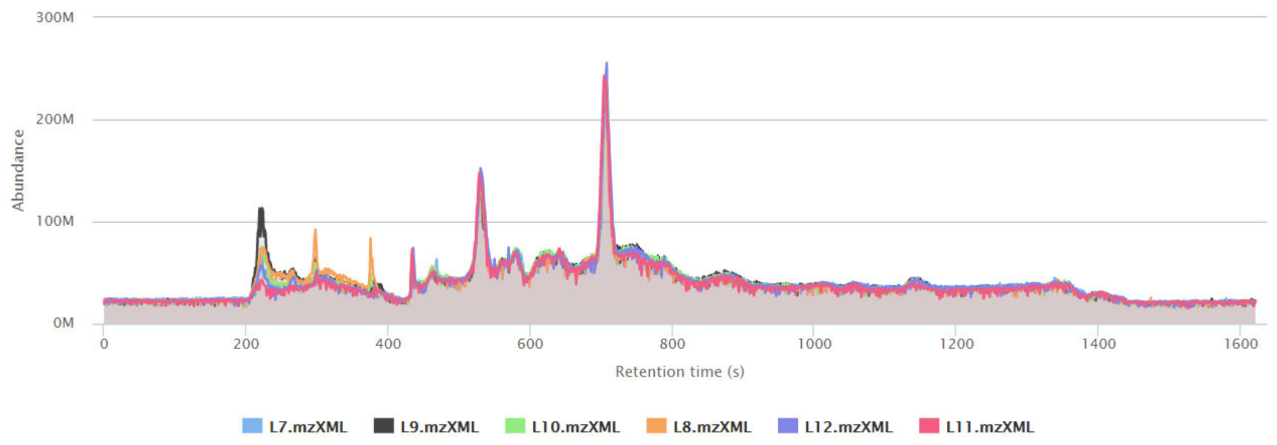

Supplementary Figure S6. Total ion chromatograms of CSF metabolites in control group (positive ion mode) generated in PiMP.

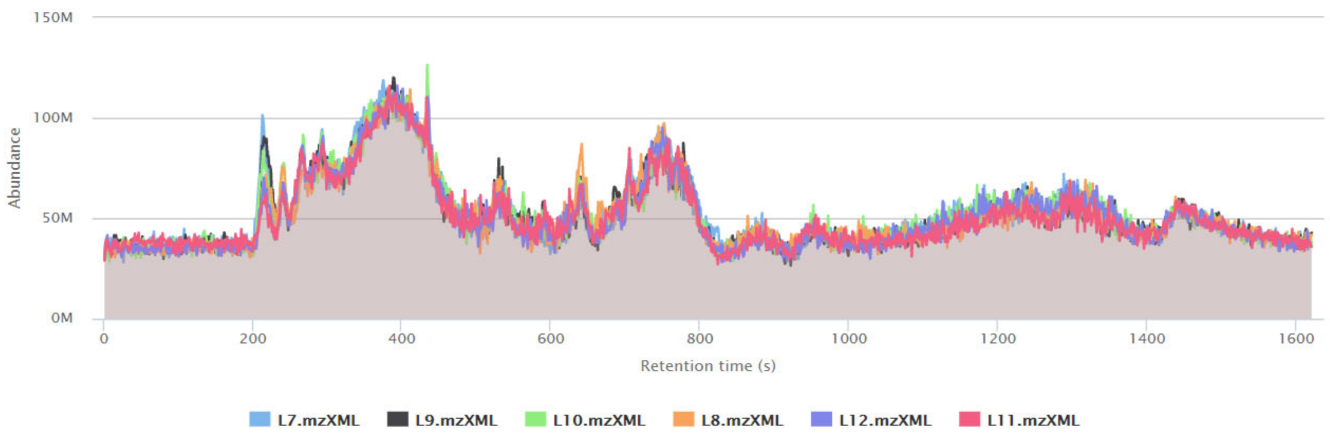

Supplementary Figure S7. Total ion chromatograms of CSF metabolites in control group (negative ion mode) generated in PiMP.

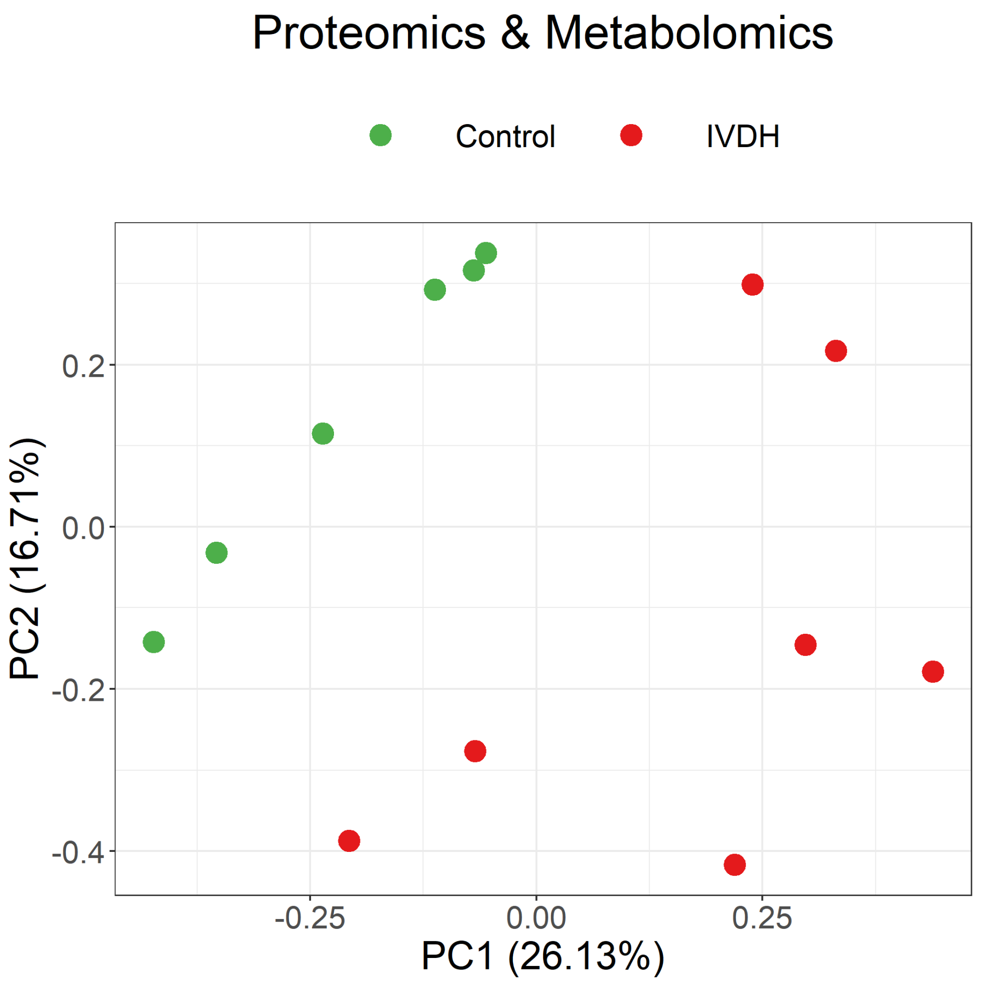

Supplementary Figure S8. Principal component analysis (PCA) of all proteomic and metabolomics features separates the control and IVDH samples when plotted against the first two principal components.

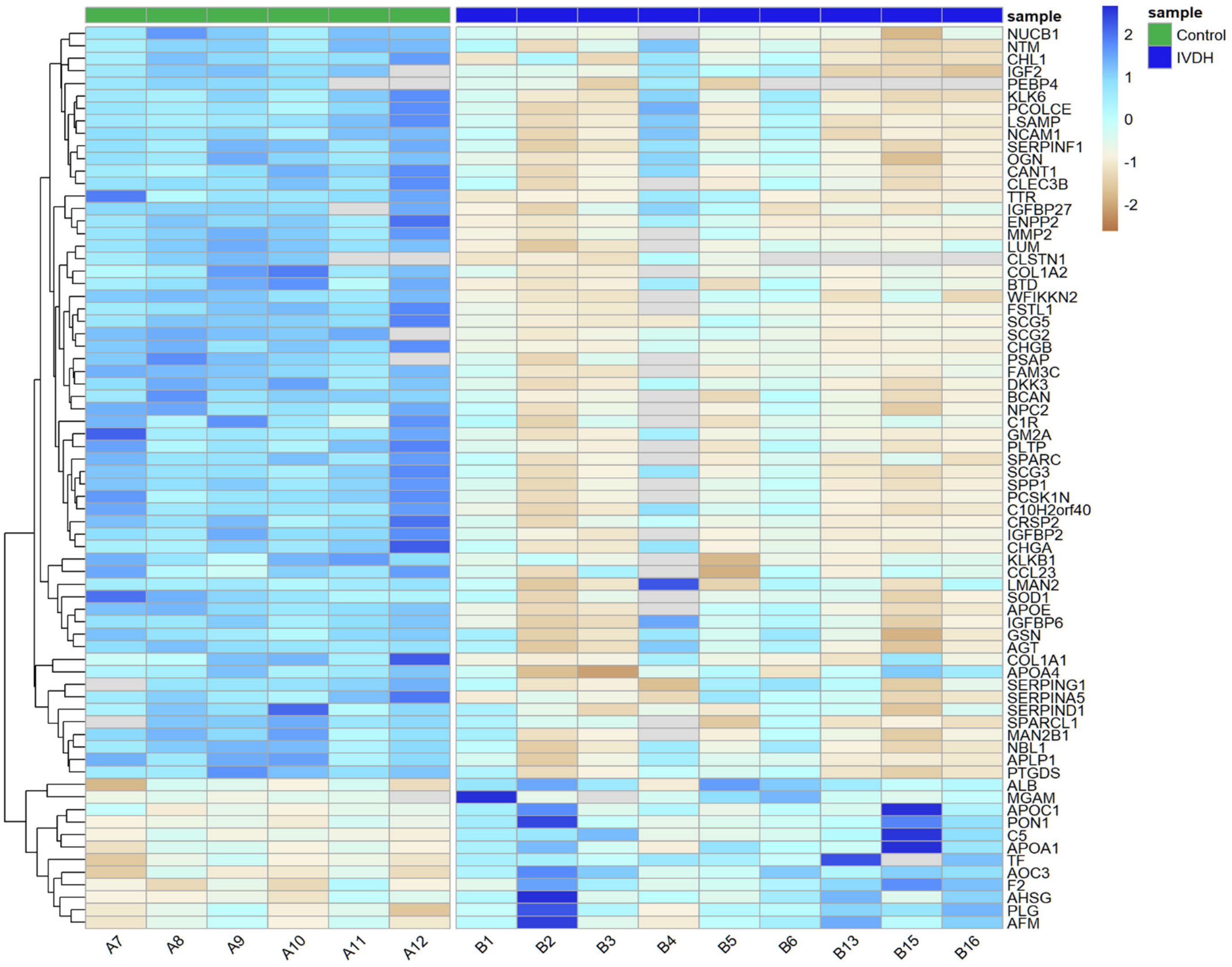

Supplementary Figure S9. (e.g. Figure 1b) Heatmap revealing the differentially expressed genes in the control and intervertebral disc herniation groups. Each row represents the fold change (FC) expressed as log2FC of a single gene across all samples. Figure 1b is also available within Supplementary files for detailed insight.

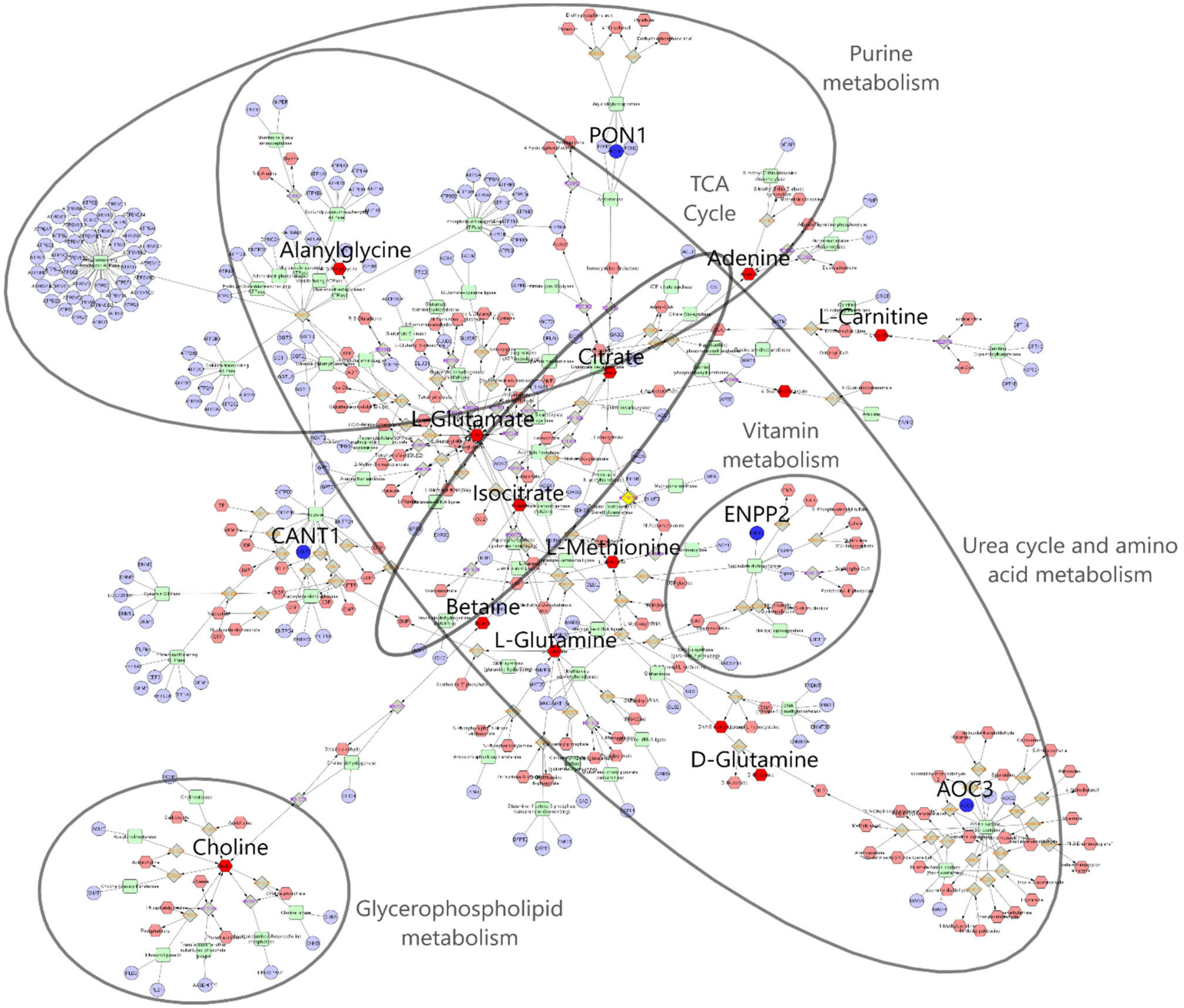

Supplementary Figure S10. (e.g. Figure 6b) Pathway-based Compound-Reaction-Enzyme-Gene (C-R-E-G) network obtained using significantly deregulated genes and metabolites visualized in Cytoscape plugin Metscape (nods: blue -

gene, red – compound/metabolite, green – enzyme). Highlighted blue (gene) and red (metabolite) nods represent input data. Enlarged feature names, together with the metabolic pathways, represent the features closely related to the IVDH. The C-R-E-G network is also available as a supplementary file for better insight into various features.
